# Metformin protects ovarian granulosa cells in chemotherapy-induced premature ovarian failure mice through AMPK/PPAR-γ/SIRT1 pathway

**DOI:** 10.1038/s41598-024-51990-z

**Published:** 2024-01-16

**Authors:** Yuxin Yang, Xiangting Tang, Ting Yao, Yiqing Zhang, Yufei Zhong, Shuqing Wu, Yurou Wang, Zezheng Pan

**Affiliations:** 1 Faculty of Jiangxi Medical College, Nanchang University, No.461 Bayi Road, Donghu District, Nanchang City, 330006 Jiangxi Province People’s Republic of China; 2 The Eighth Affiliated Hospital of Sun Yat-Sen University, Futian, Shenzhen, People’s Republic of China

**Keywords:** Biochemistry, Biological techniques, Molecular biology, Diseases, Endocrinology, Medical research, Pathogenesis

## Abstract

Premature ovarian failure (POF) caused by chemotherapy is a growing concern for female reproductive health. The use of metformin (MET), which has anti-oxidative and anti-inflammatory effects, in the treatment of POF damaged by chemotherapy drugs remains unclear. In this study, we investigated the impact of MET on POF caused by cyclophosphamide (CTX) combined with busulfan (BUS) and M1 macrophages using POF model mice and primary granule cells (GCs). Our findings demonstrate that intragastric administration of MET ameliorates ovarian damage and alleviates hormonal disruption in chemotherapy-induced POF mice. This effect is achieved through the reduction of inflammatory and oxidative stress-related harm. Additionally, MET significantly relieves abnormal inflammatory response, ROS accumulation, and senescence in primary GCs co-cultured with M1 macrophages. We also observed that this protective role of MET is closely associated with the AMPK/PPAR-γ/SIRT1 pathway in cell models. In conclusion, our results suggest that MET can protect against chemotherapy-induced ovarian injury by inducing the expression of the AMPK pathway while reducing oxidative damage and inflammation.

## Introduction

Premature ovarian failure (POF) is a type of non-physiologic amenorrhea that appears before 40 years old and has affected more than 1% of women worldwide^1,2^. Recently, increasing literature has referred to POF as premature ovarian insufficiency (POI), because its nature and its development result in a disorder of ovarian function. It is mainly characterized by an increase in follicle-stimulating hormone (FSH) and a decrease in estradiol (E2)^1^. In addition to causing disorders of hormone metabolism and female infertility, it also brings many other negative consequences to women, such as increasing the risk of cardiovascular diseases and leading to abnormal bone loss^3^. POF poses significant harm to women, and hormone replacement therapy (HRT), the commonly used clinical treatment, has been associated with numerous side effects^4^. Therefore, there is an urgent need to explore novel treatment modalities or therapeutic targets. However, at present, the etiopathogenesis of POF is not fully understood, and abnormal follicular atresia is considered to be one of the causes of POF^5^. Follicular atresia is a physiological cell death process, which generally includes oocyte apoptosis and granulosa cells (GCs) apoptosis. Studies have shown that the status of GCs is an important determinant of normal follicle initiation and development, and when GCs are in abnormal states, it may lead to POF^6,7^. Therefore, exploring the molecular mechanism of GCs proliferation or apoptosis and increasing the ability of GCs to maintain cell homeostasis can effectively prevent premature atresia of follicles, thereby prolonging the reproductive cycle and alleviating POF.

There exists abnormal GCs damage in the ovary of POF patients, but the detailed mechanism is still not established, and oxidative stress-induced GC damage is a common cause of follicular atresia^8,9^. Studies show that a larger number of reactive oxygen species (ROS) and inflammatory factors, especially IL-6 and TNF-α exist in the ovary of POF patients^10,11^, which could lead to inflammation and oxidative stress in the ovary and damage GCs. Chemotherapeutic drugs, especially cyclophosphamide (CTX), have serious side effects on the female reproductive organs, which is an important factor causing POF^12^. Ovarian toxicity caused by these drugs often disrupts the internal environment of the ovaries, leading to abnormal inflammation, oxidative stress, endoplasmic reticulum stress (ER stress), apoptosis, and other related processes^13–15^. External stimulation and internal environmental changes lead to the polarization of M0 macrophages into M1 macrophages, which can produce a large number of inflammatory factors, such as IL-6, IL-12, and TNF-α^16^, in addition, M1 macrophages also produce a large number of ROS^17^. Venkatesh, S., and Lin found that the rise of ROS is often observed in the body of POF patients^11^, and it has been reported that excessive ROS can accelerate the polarization of M0 macrophages to M1^18^. The above research indicates that the changes in the ovarian internal environment in POF patients may cause macrophages polarizing into M1 macrophages, and the secretion of inflammatory factors and ROS not only further accelerates the disturbance of the internal environment and leads to abnormal polarization of macrophages but also damages GCs, thereby accelerating follicular atresia and eventually causing POF. But further evidence is needed to support this hypothesis.

Metformin (MET) is a widely used drug for treating type 2 diabetes. Recent studies have shown that MET has various beneficial effects, including anti-aging, anti-inflammatory, and anti-cancer properties, which are mediated through the activation of AMPK (AMP-dependent protein kinase)^19^. Histone deacetylases (SIRTs) are known to respond to changes in metabolism, inflammation, and aging^20^. Among them, SIRT1 has been found to regulate GC proliferation and apoptosis, and its down-regulation has been associated with a decrease in ovarian reserve^21^. However, the detailed mechanism and pathways through which SIRT1 acts on granulosa cells have not been fully elucidated. Alam et al. proposed that SIRT1 may prevent cell dysfunction by reducing oxidative stress-induced cell damage^22^. In recent years, there have been increasing studies on the role of SIRT1 in anti-oxidative stress^22–24^, but few studies have investigated its antioxidant effect in premature ovarian failure (POF). It has been reported that the activation of AMPK can activate SIRT1 (histone deacetylase 1)^25^, additionally, PPAR-γ (peroxisome proliferator-activated receptor γ) has been reported to act as a transcription factor that inhibits SIRT1 gene expression, and the expression of PPAR-γ is inhibited by AMPK^26,27^. Although some studies have shown that MET can alleviate ovarian damage, but they have not explored the specific mechanism^28^. We consider that MET may alleviate inflammation and oxidative stress-induced damage to GCs caused by M1 macrophage through the activation of the AMPK/PPAR-γ/SIRT1 pathway.

In this study, we used MET to treat chemotherapy-induced premature ovarian failure (POF) in mice and indirectly co-cultured primary mouse GCs with M1 macrophages. Our studies aimed to investigate whether MET could alleviate GCs damage caused by inflammation and oxidative stress through the AMPK/PPAR-γ/SIRT1 pathway, thereby improving POF. And we hope this study provides a new theoretical basis and direction for clinical treatment.

## Materials and methods

### Experimental animals and treatment methods

The selection of experimental animals is an important step in animal modeling. Here, we refer to the widely used method of POF mouse modeling and adopt Cyclophosphamide (CTX) combined with Busulfan (BUS) for POF modeling. In their study, female KM mice aged 6–7 weeks were used, so we also chose this kind of mice for modeling^29^. According to the principles of animal experiment design, we included 18 mice in each experiment (a total of two animal experiments were carried out, one pilot experiment and one repeat experiment, so all available data were 36 mice). All mice were randomly divided into 3 study groups before the experiment, with 6 mice in each group. Then, these six weeks old mice were given a single intraperitoneal injection with CTX (120 mg/kg, Sigma, USA) and BUS (30 mg/kg, Sigma, USA). Four weeks later, the weight and ovarian specific gravity of the mice were significantly reduced. Most importantly, HE staining of ovarian tissue sections showed that the POF mice had an increase in ovarian atresia follicles, and changes in blood E2 and FSH levels, indicating that the model was successfully established as described in previous studies^15,29^. In our results sections “MET improved ovarian weight rate and follicular development in chemotherapy-induced POF mice” and “MET improved ovarian hormone disorder and abnormal estrous cycles in chemotherapy-induced POF mice”, we show the corresponding experimental data of successful POF model. Mice in the treatment group were given 300 mg/kg/d Metformin (MedChemExpress, China) by gavage (after 7 days of modeling, the mice received MET orally via gavage for 3 weeks) while the mice in the model group and the control group were treated with an equal volume of purified water by gavage. The determination of MET concentration was derived from prior research findings^30,31^. The laboratory mice were purchased from Laboratory Animal Center, Nanchang University. We ensured that animals had enough living space, water, as well as feed, and a sufficient 12-h day and night alternate was given. All animal experiments met the standards of the Animal Ethics Committee of Nanchang University and were reviewed and approved by the Animal Ethics Committee of Nanchang University. Besides, we confirm that any of our studies involving live animals comply with the ARRIVE guidelines^32^ and we confirm that all methods were performed in accordance with the relevant guidelines and regulations. Animal experiments were mainly performed by Yuxin Yang and Xiangting Tang. The animal research approval number is NCULAE-20221130010 and the relevant approval material can be found in the Supplementary material 1.

### Hematoxylin–Eosin staining and follicle counting

When the modeling time was over, ovaries were extracted, washed roughly with PBS, and placed in 4% paraformaldehyde overnight at room temperature for 48 h before being embedded in paraffin. After the section, fixation, deparaffinization, dehydration, and transparency, Hematoxylin–Eosin (HE) staining is carried out following the manufacturer’s instructions on hematoxylin–eosin solution staining (Servicebio, Wuhan, China). After that, the structure of the ovarian section can be observed under the light microscope, and then calculation of follicles at different levels as in previous studies^33^. Briefly, five cross-sections were randomly selected from the central region of the ovary, representing the largest cross-section. From each of these sections, five non-duplicate sections were chosen for further analysis, and the average of these five sections was calculated. The stage of the follicles was determined based on the morphology of the follicle and the granulosa cells. Following HE staining, the follicles were categorized into four groups, namely, primary follicle (PF), secondary follicle (SF), antral follicle, and atretic follicle (AF), as previously described in relevant literature^34^. The PF, whose oocytes are surrounded by a cuboidal layer of GCs. SF, with divide rapidly of granulosa cells during this period, multiple GC layers forming; Antral follicle, the most obvious feature is the appearance of follicular fluid and the formation of follicular antrum; AF, atrophy, shrinkage of follicles and cessation of development. CL, large, solid, with no visible oocytes and follicular antrum^35^.

### Analysis of estrus cycle by Swiss-Giemsa staining

Every day at 10:00 am, we conducted vaginal smears to assess the estrous cycle of model mice for a consecutive 7-day cycle, which lasted for a total of 14 days since the mice were given modeling medication for 2 weeks. On the operating table, we rinsed the vagina of the mice with 10 μL of double-distilled water, and then collected the fluid and placed it on a slide. Generally speaking, after natural drying and using Swiss-Giemsa staining, according to the type and proportion of cells in the vaginal fluid (especially leukocytes, nucleated epithelial cells, and cornified squamous epithelial cells) that had been just collected by an optical microscope, we may infer which estrous phase the mice was in. Normally, the estrous cycle consists of 4 phases, including proestrus, estrus, metestrus, and diestrus^36^. Proestrus smears were dominated by nucleated epithelial cells, while estrus smears were predominantly composed of nucleated keratinocytes. Metestrus smears were composed of leukemic cells, keratinocytes, and nucleated epithelial cells in equal proportions, whereas diestrus smears are predominantly leukocytes.

### Hormone measurement with ELISA

After modeling, serum samples were collected from the eyeball vein, and allowed to coagulate naturally for 20 min at room temperature, and the upper layer of serum was carefully collected after centrifugation at 3000 rpm, 4 °C for 15 min. We used ELISA kits to measure the levels of FSH and E2 in the serum samples (E-EL-M0511c, Elabscience, Wuhan, China; E-OSEL-R0001, Elabscience, Wuhan, China). In short, samples were added to the enzyme-coated plates in turn, then incubated at 37 °C for 30 min with plate sealing membrane. Next, washed 3–5 times, followed by the addition of enzyme-labeled reagents, continued incubation for 30 min, and then washing. Finally, chromogen was added and the reaction was shielded from light for 15 min. OD was measured at a wavelength of 450 nm after the addition of the termination solution.

### Primary ovarian granulosa cell isolation and culture

According to the method of Tang and Shen^15,37^, we employed a mechanical method for the extraction of ovarian granulosa cells (GCs). In brief, the ovaries were collected from 3-4 weeks mice, until 46–48 h after the intraperitoneal injection of 20U pregnant mare serum gonadotropin (PMSG), then the follicles were continuously punctured using a 1 ml syringe needle under a dissecting microscope (the elevated, translucent surface of the ovary, seen microscopically, shows a large number of follicles containing GCs). Subsequently, centrifugation at 1200r/min for 5 min was performed to collect. Then the cells were placed in a culture plate with DMEM/F12 (Gibco, USA) medium containing 15%FBS (Gibco, USA) and 1% antibiotics and incubated them at 37 °C with 5% CO_2_. After 12–16 h, it was necessary to change the medium. During this period, most of the GCs could adhere to and survive, while other miscellaneous cells were eliminated. The culture was continued under the same conditions for 24–48 h, then when the cells have grown to a suitable density, the next step of the experiment can be started. The successful isolation and culture of GCs were confirmed through identification as described in Section Results-“Co-culture of M1 macrophages supernatant with primary GCs caused abnormal inflammatory response, ROS accumulation, and senescence”. After incubation reached the desired number of cells, different cell models can be processed, for example, when the experiment was divided into CON group, GCs co-culture M1 supernatant, GCs co-culture M1 + MET, and MET alone. The old medium in the six-well plate was discarded, a new 1 ml of the medium was added, and then one well of the six-well plate was co-incubated with 1 ml M1 supernatant for 24 h. In the GCs co-culture M1 + MET group, 1 mM MET was added immediately after 1 ml M1 supernatant was added, and 1 mM MET for 24 h. The concentration of MET was based on previous studies^30,38^.

### Macrophage culture and treatment

We purchased RAW264.7 cell lines from Shanghai Cell Bank, Chinese Academy of Sciences, Following the guidelines for the official website, we use the special culture medium of RAW264.7 cell (TCM-G766, cas9x, China) to cultivate them. When the cells grew to the desired density, 100 ng/ml LPS was added to the culture for 24 h (the concentration and time were based on previous studies^39^). After successful polarization, obvious changes in cell morphology and growth habits could be observed (in Supplementary Materials 2), and corresponding biomarkers (CD86, CD206 and so on) could be detected (in Results section “Co-culture of m1 macrophages supernatant with primary gcs caused abnormal inflammatory response, ros accumulation, and senescence”). For the supernatant of M1 macrophages, after successful polarization by adding LPS, the medium containing LPS was discarded and a new medium was added to continue the incubation for 24 h. After 24 h, the supernatant was collected by centrifugation and added to primary GCs to achieve indirect co-culture.

### Cell immunofluorescence

After the GCs were extracted from the mice, they were cultured in a 3.5 cm petri dish (5% CO_2_, 37 ℃). Firstly, we fixed the GCs with 4% paraformaldehyde for 10–15 min after the cells reached the appropriate density (50–60%) to ensure a staining effect. Before the next step, for the antibody to bind smoothly, 1% Triton X-100 was used to appear cells for 15 min, and then PBS was washed three times, each time for 5 min. After that, the cells by 1% BSA at room temperature lasting 2 h. Next, incubate with FSHR primary antibody (1:200) (Wuhan Protein Technology Co., LTD., China) overnight at 4 °C, which was followed by 3 times washing by PBS the second day. With DAPI staining the nucleus for 5 min followed by PBS washing three times, we subsequently incubated GCs with AffiniPure Goat Anti-Rabbit IgG (H + L) (Boster, Wuhan, China) at room temperature for 1 h. Finally, we captured cells with successful color development under fluorescence microscopy.

### ROS level measurement by DCFH-DA

To evaluate the intracellular ROS levels, we used a ROS Assay kit (Beyotime, S0033S, China). Firstly, we seeded GCs in a 6-well plate, and after the cells had grown to a suitable density, M0 supernatant, M1 supernatant, metformin, and other treatments were added as described previously. Next, following kit instructions, remove the medium, with 10 μM DCFH-DA serum-free medium in 37 ℃ incubation GCs for 30 min, after that use PBS washing 3 times. Finally, ROS levels were immediately measured at a wavelength of 488 nm in the microscope and the positives are shown in green.

### Senescence-associated β-Galactosidase staining

To measure the senescence activity in the GCs, we used the Senescence-associated β-Galactosidase Staining kit (Beyotime, C0602, China) following the manufacturer’s instructions. Briefly, seeded GCs in a 6-well plate in an appropriate density. Then it was treated with M0 supernatant, M1 supernatant, Metformin, etc. in different groups. When the processing time was reached, GCs were fixed with 4% paraformaldehyde for 15 min and then washed with PBS 3 times. Eventually, add β-Gal substrate and according to the instructions incubate overnight at 37 °C. Subsequently, after three washings with PBS, the positive senescent cells appeared blue-green with a light microscope. The degree of senescence was proportional to the number of blue-green cells.

### qPCR

We used TRIzol reagent (Takara, Japan) to extract total RNA from ovary tissue or cell samples, and approximately 400 μl of TRIzol was added to a 6-well plate of primary granulosa cells, while 1 ml of TRIzol was added to 100 mg of tissue. According to the instructions of the PrimeScript RT kit (Japanese Takara) to reverse mRNA, cDNA was obtained for further qPCR. We used a real-time PCR supermix kit (Takara, Japan) and specific primers to conduct the amplification of cDNA. The relative mRNA levels were determined using the 2^−ΔΔCT^ method^40^, with β-actin (ACTB) serving as the reference gene. The primer sequences used for PCR amplification of specific gene segments are provided in Table 1.

**Table 1:** Sequences used for quantitative real-time PCR.

Gene name	Primer sequence: 5′-3′
ACTB	Forward: CTACCTCATGAGATCCTGACC Reverse: CACAGCTTCTCTTTGATGTCAC
AMH	Forward: GGGCTCTAAGCGCCTATGAG Reverse: TCCACCGCTAACACCAGGTA
CD206	Forward: TTCAGCTATTGGACGCGAGG Reverse: GAATCTGACACCCAGCGGAA
CD86	Forward: TGTGATCTTCGGGAATGCTGC Reverse: TCTCCACGGAAACAGCATCTGAG
ARG-1	Forward: ATCGGAGCGCCTTTCTCAAA Reverse: CTTCCAACTGCCAGACTGTG
INOS	Forward: TCTAGTGAAGCAAAGCCCAACA Reverse: TGATGGACCCCAAGCAAGAC
IL-6	Forward: CAACGATGATGCACTTGCAGA Reverse: TGTGACTCCAGCTTATCTCTTGG
IL-10	Forward: GGTTGCCAAGCCTTATCGGA Reverse: CACCTTGGTCTTGGAGCTTATT
TNF-α	Forward: ACCCTCACACTCACAAACCA Reverse: ATAGCAAATCGGCTGACGGT
CAT	Forward: AAGATTGCCTTCTCCGGGTG Reverse: GACATCAGGTCTCTGCGAGG
SOD2	Forward: AGGAGAGTTGCTGGAGGCTA Reverse: AGCGGAATAAGGCCTGTTGTT
GPX	Forward: TTGGTCATTCTGGGCTTCCC Reverse: AGGGCAGGAGTTCTTCAGGA

### Western blotting

To conduct Western blotting (WB), the corresponding ovary tissues or cells were treated with RIPA lysis buffer (Applygen, Beijing, China) to extract proteins at low temperatures. Then we used the BCA kit (Applygen, China) to quantitatively detect the extracted proteins so that the internal parameters of the proteins in Western Blotting were equal and the reliability of the experimental data was enhanced. After being separated by SDS–polyacrylamide gel electrophoresis (80 V, constant pressure) and transferred to a PVDF membrane (Millipore, Darmstadt, Germany) (200A, constant current, 1.5–2 h), the sample proteins were imprinted on the membranes. Then we blocked the membranes with a 5% skim milk powder for 2 h (or rapid blocking solution, 30 min). After that incubate the membranes with primary antibodies overnight at 4 °C. The primary antibodies used in this study can be seen in the Table 2 below. Except for the dilution ratio of 1:10,000 for ACTB, the dilution ratio of the other antibodies was 1:1000 to 1:5000. The PVDF membranes were cut before hybridization with a single specific target antibody during blotting for better imaging. For some antibodies with poor specificity and more miscellaneous bands, whether they were the target protein was judged according to the prestained protein marker. If the imaging effect is too poor to distinguish two target proteins that are very close to each other, they can only be repeated once and incubated separately. The corresponding secondary antibodies (Affinity Biosciences, Cincinnati, USA) were incubated for 1 h at room temperature and then we used the Super ECL Detection Reagent (36208ES60, Yeasenbiotech, China) to make the blot visualize. The images were excited by ultraviolet light and scanned using Image Lab, which was quantified using Image J. Attention, to prevent any negative effect to the following incubation, after each antibody incubation, the membranes were washed three times with TBST, each for 5–10 min. The images were scanned and analyzed using Image Lab and Image J. We declare and ensure that our WB data comply with the digital image and integrity policies.

**Table 2:** The Company and Catalogue number of all primary antibodies.

Name	Observed molecular weight	Company and Catalogue number
ACTB	42 kDa	Proteintech, China (81115-1-RR)
BCL2	26 kDa	Proteintech, China (68103-1-Ig)
BAX	21 kDa	Proteintech, China (60267-1-Ig)
CD86	60–80 kDa	Proteintech, China (13395-1-AP)
CD206	170 kDa	Proteintech, China (18704-1-AP)
P53	53 kDa	Proteintech, China (60283-2-Ig)
P21	21 kDa	Proteintech, China (10355-1-AP)
IL-6	24 kDa	HUABIO, China (R1412-2)
TNF-α	17–26 kDa	WanleiBio, China (WL01581)
SIRT1	110–130 kDa	Proteintech, China (60303-1-Ig)
PPAR-gamma	50–58 kDa	Proteintech, China (66936-1-Ig)
AMPKα	62 kDa	Cell signaling, America (5832)
p-AMPKα	62 kDa	Cell signaling, America (2535)

### Statistical analysis

The experimental data needed for statistics and analysis were quantified from a minimum of three independent experiments. After processing the experimental data using the appropriate software, the data was imported to GraphPad Prism 9.0.0 for analysis. The software’s built-in features were utilized to perform data analysis operations on different datasets. One-way analysis of variance (ANOVA) was employed as the primary statistical method in this experiment. Subsequently, the results were presented using bar charts or line charts. The p values were labeled on the charts, and corresponding visual statistical images were generated. Statistical significance was determined for *p* values less than 0.05, denoted as (* for *p* < 0.05, ** for *p* < 0.01, and *** for *p* < 0.001).

## Results

### MET improved ovarian weight rate and follicular development in chemotherapy-induced POF mice

**Figure 1:**
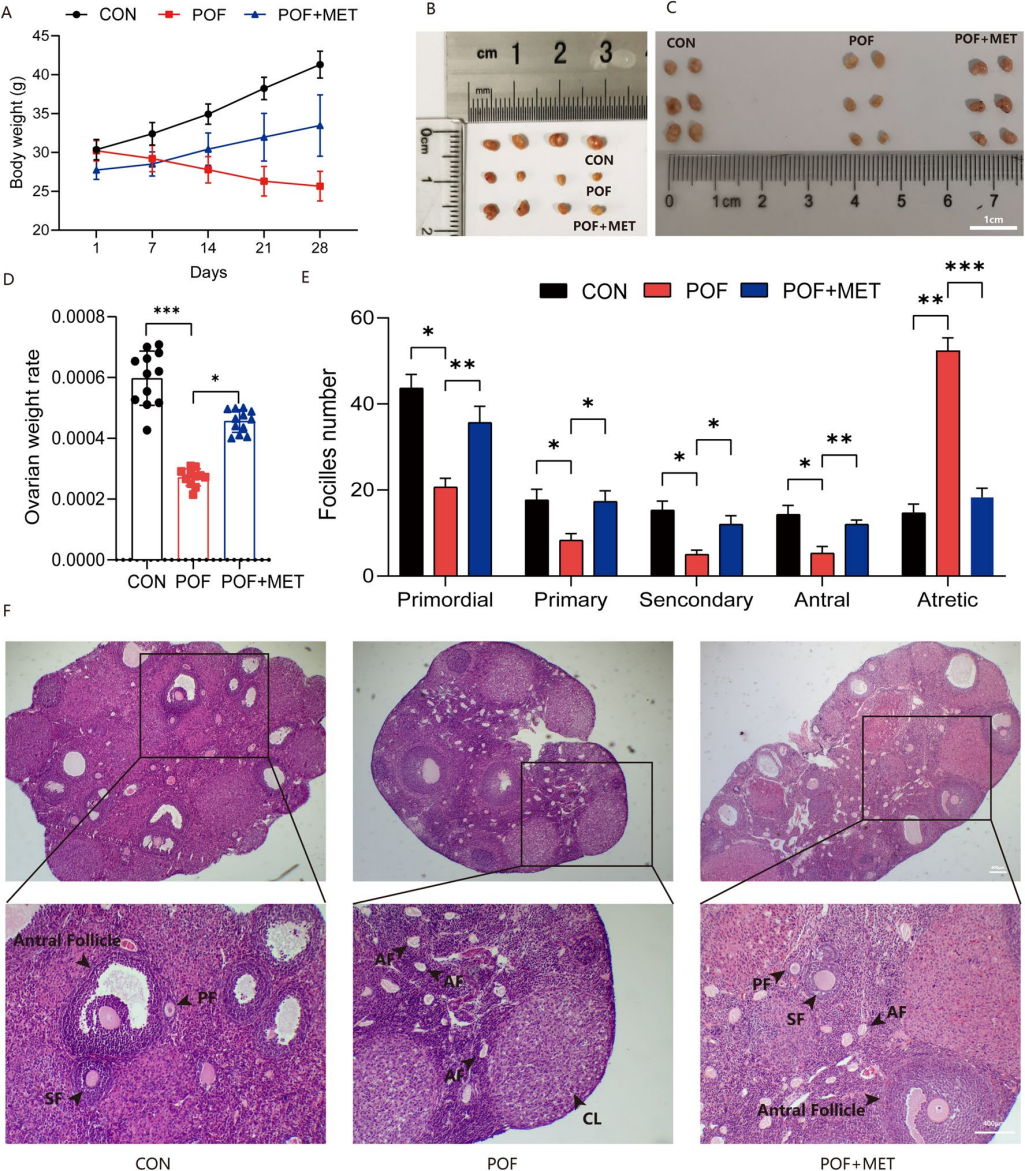
MET improved ovarian specific gravity and follicular development in POF mice. (**A**): The weights of mice every week in different treated groups. (**B**,**C**): Ovarian tissues from five mice per treatment group were displayed (scale bar: 1 cm). (**D**): The ovary/mouse relative weight ratio in control, POF, and MET groups after 4 weeks of treatment, individually. (**E**,**F**): After HE staining, the structure of follicles at all levels was observed under a 40× and 100× microscope (scale bar: 400 μm), and the number of follicles in different stage (Primary follicle (PF), Secondary follicle (SF), Atresia follicle (AF), Corpus Luteum (CL)) was counted by tissue sections. (*p < 0.05, **p < 0.01, ***p < 0.001).

Lavage was performed on the POF model mice with 300 mg/kg/d MET for 3 weeks after injecting CTX + BUS for one week as described before (Section “Experimental animals and treatment methods”). Then, the mouse weights, ovarian weights, and ovary/mouse relative weights were calculated, individually, body weight was measured every 7 days, and mouse ovaries were removed after 28 days. Figure 1A–D showed that the body weight of POF mice were decreased and ovarian volume was atrophic. However, after gavage of MET to POF mice, the body weight was gradually increased, and the ovarian volume and weight were recovered (*p* < 0.05); In Fig. 1E and F, the number of primordial follicles and growing follicles at all levels decreased to varying degrees, the number of atretic follicles increased significantly, and the ovarian function was significantly impaired, indicating that the model was successful. However, the number of growing follicles at all levels tended to be normal after MET administration (*p* < 0.05), indicating that MET improved abnormal follicular development in POF mice.

### MET improved ovarian hormone disorder and abnormal estrous cycles in chemotherapy-induced POF mice

**Figure 2:**
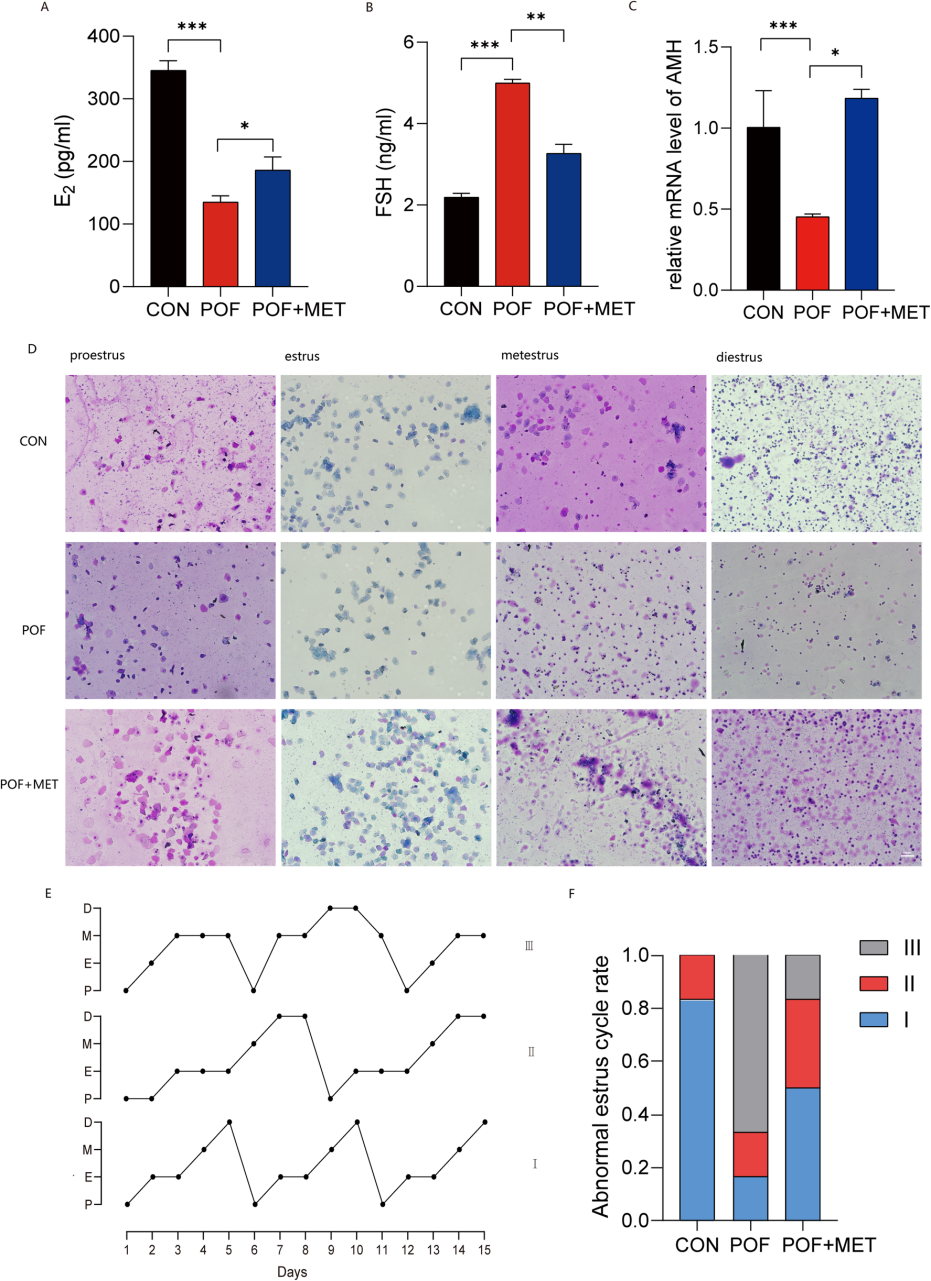
MET improved ovarian hormone disorder and abnormal estrous cycles in chemotherapy-induced POF mice. (**A**,**B**): The results of serum E2 and FSH ELISA experiments. (**C**): The qPCR experiment of the ovarian reserve indicator AMH. (**D**): The results of some vaginal smears were shown under the microscope (scale bar: 100 μm). (**E**): Three types of estrus rules, I means the normal estrous cycle, II shows prolonged estrous cycle and III, irregular estrous cycle disorder. (**F**): Ratio of three types of estrous cycles in each treatment group.

Figure 2A and B present the results of serum enzyme-linked immunosorbent assay (ELISA) experiments. These findings demonstrate that the administration of MET to POF mice results in a significant increase in the concentration of E2 (*p*  < 0.05) and a noteworthy decrease in the concentration of FSH (*p* < 0.01) when compared to the POF group. Figure 2C represents the qPCR experiment of the ovarian reserve indicator Anti-Mullerian Hormone (AMH), which displayed a decrease in the expression of AMH in POF mice and an increase after MET administration (*p* < 0.05). These results indicate an improvement in the hormonal disorder in mice. Additionally, vaginal smears were performed every day for 14 consecutive days after 7 days of MET treatment in POF mice, revealing that the POF group had a lower number of cells in estrus (large polygonal cornified squamous epithelial cells), whereas MET treatment effectively restored the number of cells in estrus (Fig. 2D), and the regularity of estrus was recorded. Figure 2E illustrates the line chart depicting the estrous regularity in the three groups of mice. In this chart, ‘III’ represents the normal estrous cycle of mice, which lasts 4–6 days and includes 1–2 days in diestrus, 1 day in proestrus, 1 day in estrus, and 1 day in diestrus^41^. ‘II’ indicates a prolonged estrous cycle, while ‘I’ suggests an irregular estrous cycle disorder. Figure 2F presents the ratio of the three types of estrous cycles in the three groups of mice, demonstrating that MET treatment effectively improved the abnormal estrous cycle in POF mice.

### MET alleviates the abnormal inflammatory response, oxidative stress, and apoptosis in the ovaries of chemotherapy-induced POF mice

**Figure 3:**
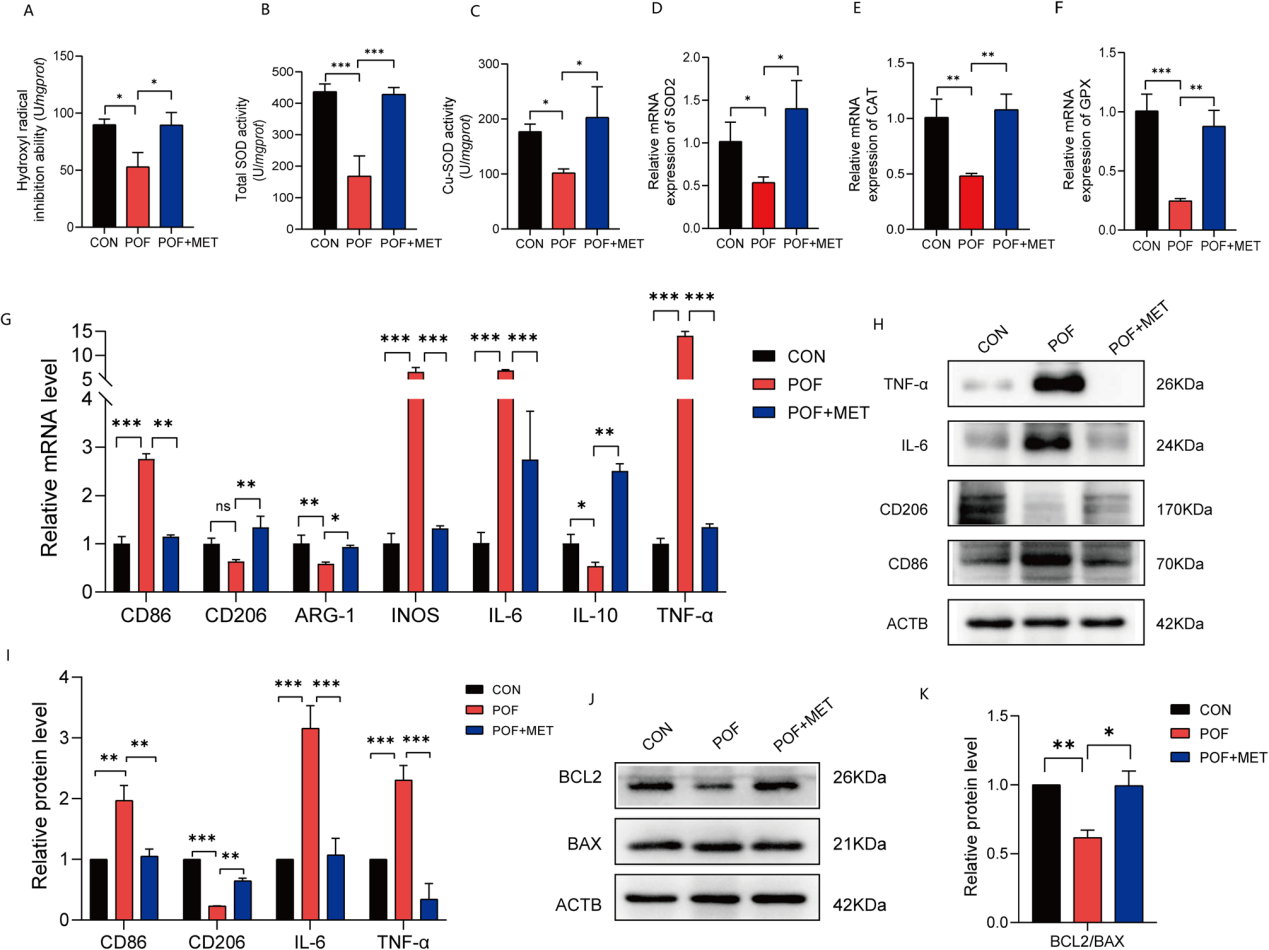
MET alleviates the abnormal inflammatory response, oxidative stress, and apoptosis in the ovaries of chemotherapy-induced POF mice. (**A**–**C**): Measurement of antioxidant enzyme activity. (**D**–**F**): Measurement of antioxidant enzyme expression by qPCR. (**G**): The results of qPCR to detect macrophage surface markers as well as some inflammatory factors. (**H**,**I**): The results of the western blot to detect macrophage surface markers and some inflammatory factors. (**J**,**K**): The results of western blot to detect apoptosis-related molecules.

Catalase (CAT), glutathione peroxidase (GPX), and superoxide dismutase (SOD) are essential components of the enzymatic antioxidant system^42^. The activities of antioxidant enzymes were assessed in Fig. 3A–C. Compared to the POF group, MET administration led to increased OH-scavenging activity, as well as increased total SOD activity and Cu-SOD activity (*p* < 0.05) qPCR results revealed that the expression of antioxidant enzymes CAT, GPX, and SOD2 mRNA was lower in the POF group compared to normal mice (*p* < 0.05). However, the expression of antioxidant enzymes in ovarian tissues was upregulated in MET-treated mice (*p* < 0.05) (Fig. 3D–F). These findings suggest that chemotherapy-induced POF mice demonstrate reduced ovarian antioxidant activity and expression. However, after administration of MET, the antioxidant activity and expression were increased, indicating that MET enhances the antioxidant capacity of ovarian tissues. Figure 3G–I present the results of qPCR and western blot analysis, indicating that the expression of M1 macrophage molecular markers CD86 and INOS was increased, while the expression of M2 macrophage markers CD206 and ARG-1 was decreased^16^. Additionally, the secretion of inflammatory cytokines IL-6 and TNF-α was increased (*p* < 0.05). These findings suggest the presence of M1 macrophage polarization and abnormal accumulation of pro-inflammatory cytokines in the ovaries of chemotherapy-induced POF mice. However, after administration of MET, the altered molecules in POF mice showed opposite changes, indicating that MET alleviated the abnormal ovarian inflammatory response and inhibited the polarization of macrophages towards the M1 phenotype to a certain extent. Besides, we investigated apoptotic molecules (Fig. 3J,K) and observed that the ratio of BCL2/BAX was up-regulated, indicating increased anti-apoptotic ability following MET treatment (*p* < 0.05).

### Co-culture of M1 macrophages supernatant with primary GCs caused abnormal inflammatory response, ROS accumulation, and senescence

**Figure 4:**
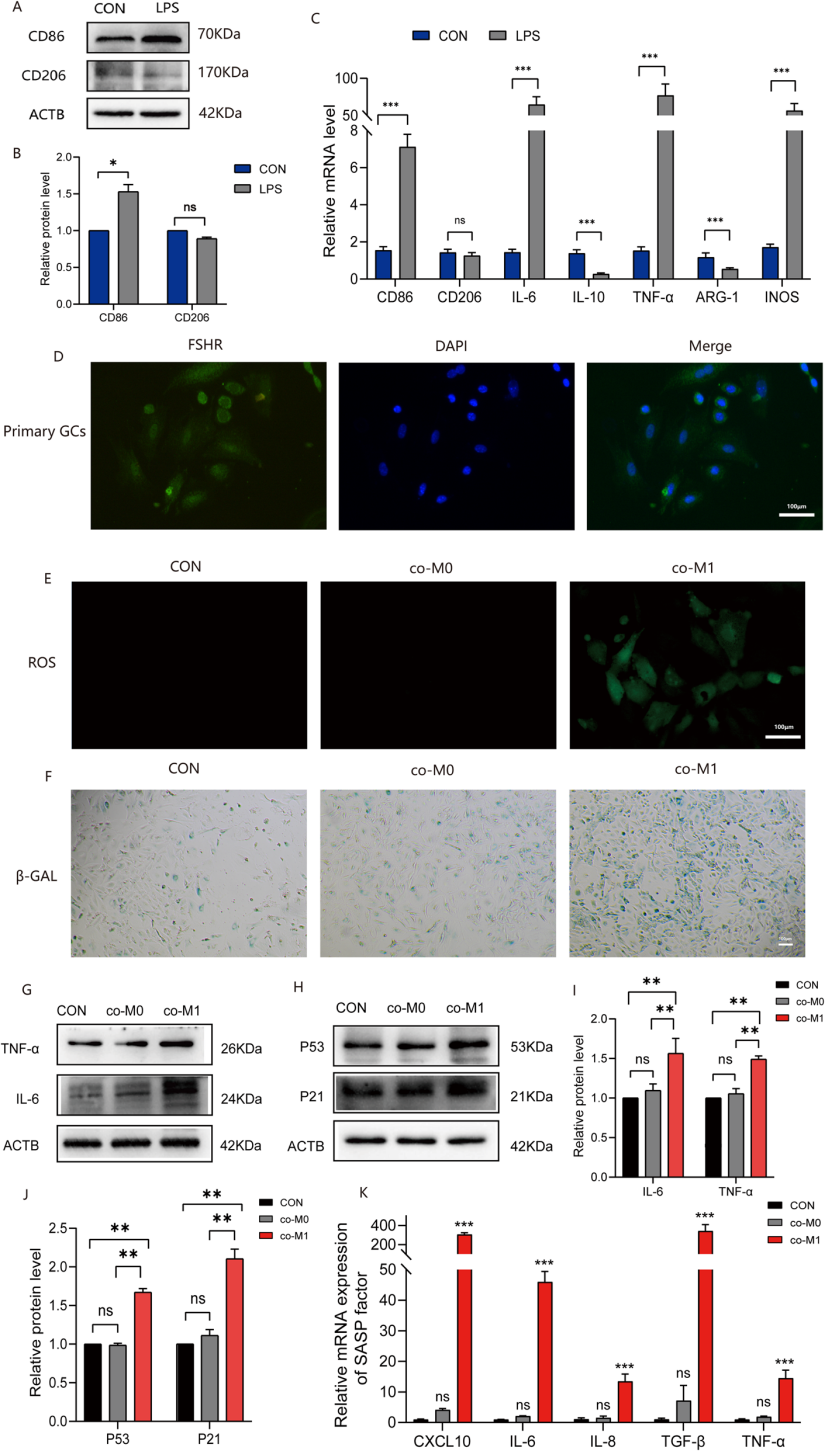
Co-culture of M1 macrophages supernatant with primary GCs caused abnormal inflammatory response, ROS accumulation, and senescence. (**A**–**C**): The identification of M1 macrophages. (**D**): The identification of primary GCs (scale bar: 100 μm). (**E**): The results of ROS level measurement (scale bar: 100 μm). (**F**): The results of senescence-associated β-Galactosidase staining (scale bar: 100 μm). (**G**–**J**): The results of WB to detect inflammation and senescence-related molecules. (**K**): The results of qPCR to detect SASP factors.

The method for polarization of RAW264.7 cells into M1 macrophages was described in section "Analysis of estrus cycle by Swiss-Giemsa staining" of the previous text. Successful polarization was confirmed by obvious changes in cell morphology (We present two pictures and describe this morphological change in supplementary material 2). In addition, to validate polarization at the molecular level, we examined the expression of surface molecular markers on M1 and M2 macrophages. CD86 and iNOS were identified as molecular markers for M1 polarization, while CD206 and ARG-1 were identified as molecular markers for M2 polarization as confirmed by previous research^16^. Western blot analysis (Fig. 4A,B) revealed a significant upregulation of CD86 expression following LPS-induced polarization (*p* < 0.05), however, no statistically significant changes were observed in CD206 expression. Figure 4C demonstrates that upon induction with 100 ng/mL LPS, the expression of M1 macrophage markers and some pro-inflammatory cytokine mRNA increased significantly (*p* < 0.05). Figure 4D presents the immunofluorescence results to identify primary GCs, with the GC surface marker FSHR labeled in green, thus confirming the presence of ovarian GCs. These results confirm the identification of M1 macrophages and primary GCs. Figure 4E and F depict the results of ROS staining and β-GAL staining, respectively. It was observed that in the co-M1 group, there was an increase in green fluorescent ROS staining and an increase in blue-green senescent cells. On the other hand, no significant changes were observed in the co-Mo group. These findings indicate elevated levels of ROS and senescence in CC after co-culture with M1 macrophages. Furthermore, we employed WB and qPCR experiments (Fig. 4K) to investigate the levels of inflammatory molecules IL-6 and TNF-α (Fig. 4G,I), as well as senescence marker proteins P53 and P21(Fig. 4H,J), at the molecular level. Our results demonstrated that the indirect co-culture of primary GCs with M1 macrophage resulted in an elevation in the accumulation of inflammatory factors, oxidative stress, and senescence of primary GCs.

### MET significantly mitigated the damage inflicted on primary GCs which are co-cultured with M1 macrophages

**Figure 5:**
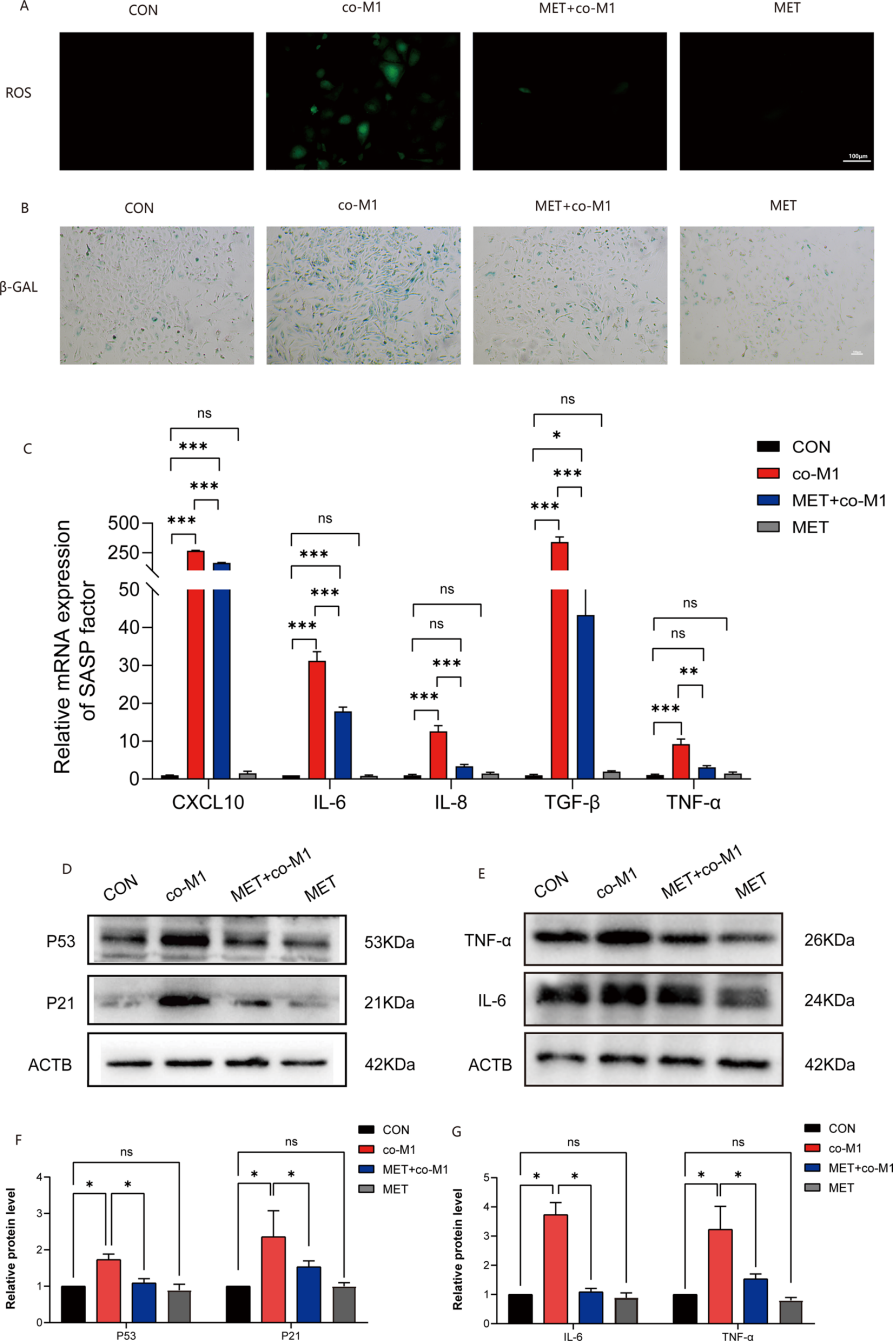
MET significantly mitigated the damage inflicted on primary GCs which are co-cultured with M1 macrophages. (**A**): The results of ROS level measurement (scale bar: 100 μm). (**B**): The results of senescence-associated β-Galactosidase staining (scale bar: 100 μm). (**C**): The results of qPCR to detect SASP factors. (**D**–**G**): The results of WB to detect inflammation and senescence-related molecules.

As previously mentioned, it has been well-established that co-culture of M1 supernatant with GCs leads to impaired functioning, characterized by significant accumulation of ROS, increased senescence, and inflammation, in comparison to primary GC co-culture with M0 supernatant. In this cell experiment, M1 supernatant was added to primary GCs, followed by an immediate addition of 1 mM MET, and then cultured for 24 h to observe whether there were any changes in the injury of GCs after co-culture with M1. The results of Fig. 5A, stained with AROS, demonstrated a significant reduction in ROS accumulation upon the addition of MET. Similarly, the results of the aging staining revealed that MET could improve the aging of damaged GCs (Fig. 5B). Moreover, we observed a significant increase in the levels of SASP factors after treatment with M1 supernatant, while these indicators significantly decreased after treatment with MET (*p* < 0.05) (Fig. 5C). Next, we also examined the levels of aging-related molecules P53, P21 (Fig. 5D,F), as well as inflammation-related molecules TNF-α and IL-6 (Fig. 5E,G). The results demonstrated that the addition of MET significantly improved both the aging and inflammation of GCs. These findings indicate that MET plays a significant role in mitigating the damage inflicted on primary GCs that are co-cultured with M1 macrophages.

### MET alleviates GCs injury induced by M1 macrophages through the AMPK/PPAR-γ/SIRT1 pathway

**Figure 6:**
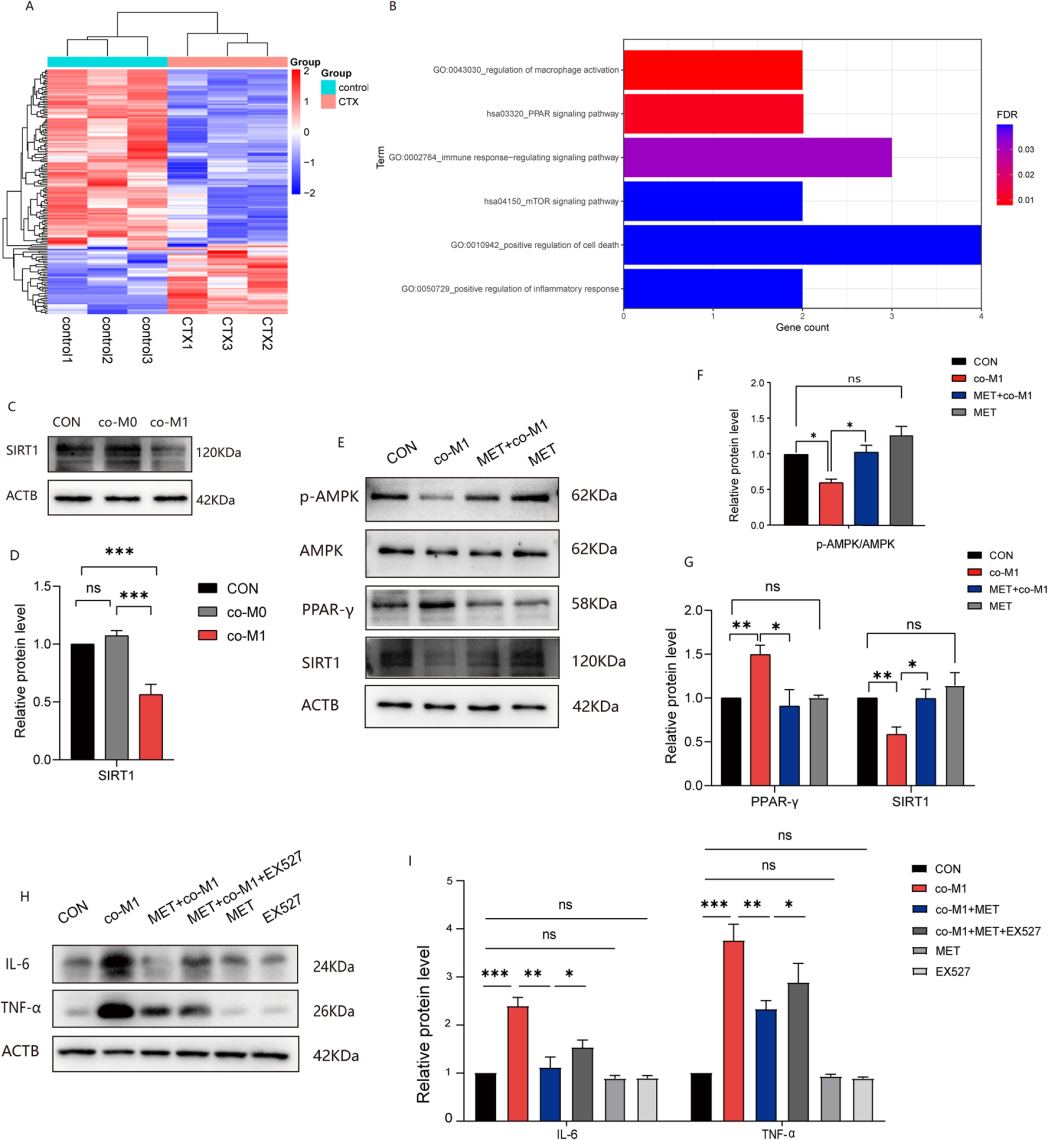
MET alleviates GCs injury induced by M1 macrophages through the AMPK/PPAR-γ/SIRT1 pathway. (**A**,**B**): The results of bioinformatics analysis. (**C**–**I**): WB and quantification results of different treatment groups.

In this part, we conducted bioinformatics analysis using the GEO database, GSE128240 dataset. Our analysis revealed significant differences in the expression of genes between healthy mouse ovaries and ovaries from chemotherapy-induced POF mice (Fig. 6A). Further analysis focused on the differentially expressed genes and their enrichment in relevant functional pathways through KEGG analysis, revealing signaling pathways like cell death, immune response, and PPAR pathways were enriched (Fig. 6B). In Fig. 6C,D, our results demonstrated a significant decrease in SIRT1 expression in the co-M1 group, implying a reduction in the antioxidant capacity of GCs under inflammatory exposure induced by M1 supernatant treatment. Subsequently, we administered MET to the co-M1 group. WB analysis of AMPK, p-AMPK, PPAR-γ, and SIRT1 revealed that the ratio of p-AMPK/AMPK decreased in the co-M1 group (Fig. 6E–G), and this ratio partially recovered following MET administration (*p* < 0.05). This indicates that MET can activate AMPK, while treatment with M1 supernatant inhibits AMPK expression in primary GCs. Additionally, PPAR-γ exhibited an opposite trend to the p-AMPK/AMPK ratio, and SIRT1 showed a similar trend to the p-AMPK/AMPK ratio in different treatment groups. These findings suggest that MET may enhance SIRT1 expression in GCs through the AMPK/PPAR-γ/SIRT1 pathway, leading to an improved antioxidant capacity. Furthermore, to assess whether blocking SIRT1 alters the therapeutic effect of MET, we pretreated cells with EX527, a SIRT1 inhibitor, following the concentration and duration described in previous studies^43^. We observed that IL-6 and TNF-α levels in the co-M1 + MET + EX527 group were higher than those in the co-M1 + MET group (Fig. 6H,I), indicating that blocking SIRT1 diminishes the anti-inflammatory effect of MET on GCs. These additional results further support the notion that MET enhances the anti-inflammatory and anti-oxidative capacity of GCs through the AMPK/PPAR-γ/SIRT1 pathway, ultimately improving GCs' ability to maintain cellular homeostasis.

## Discussion

Increasing evidence suggests that abnormal inflammatory states and oxidative stress in GCs are important causes of the decline in ovarian reserve^9,44,45^ and many antioxidant drugs have been explored for their potential to protect against POF^13,46^. MET, as a multifunctional drug that has been extensively studied in recent years, has been shown to have anti-inflammatory, antioxidant, and anti-aging effects in various disease models^47,48^. The function of MET is continuously being explored and discovered, in recent years, numerous studies utilizing different cell lines and models have indicated the tremendous potential of MET in delaying aging and alleviating age-related diseases by targeting key molecules associated with aging^49^. For instance, in cardiovascular diseases, early chronic oral administration of nitrite and MET can reduce pulmonary pressure and vascular remodeling. This effect at least partially involves the activation of skeletal muscle SIRT3-AMPK and improvement in glucose uptake and metabolism^50^. In metabolic disorders such as fatty liver disease, MET improves fatty liver by inhibiting the expression of TNF-α in the liver, suppressing lipid accumulation, and reducing ATP consumption, leading to the reversal of hepatomegaly, lipid degeneration, and abnormal liver enzymes^51^.

Furthermore, MET has also been extensively used in the field of reproductive medicine^52^, specifically for the treatment of polycystic ovary syndrome (PCOS), and its use has been included in clinical guidelines^53^. However, there is a lack of research on its effects on GCs in chemotherapy-induced POF. In the past, research in the field of reproduction has focused more on the effect of MET on the ovary in PCOS, but in recent studies, we found that there have been studies showing that MET can alleviate ovarian damage, such as study by Du et al.^28^ in 2022 showed that metformin, dasatinib, and quercetin, alone or in combination use can significantly improve ovarian function and alleviate age-related secretory phenotypes. This study suggested the possibility of MET alleviating premature ovarian failure and restoring ovarian function, but unfortunately, he did not investigate the specific mechanism of action of MET. In October 2023, a study of MET by Chen et al.^54^ showed that metformin can promotes proliferation of mouse female germline stem cells, and their study shows that to a certain extent, metformin is beneficial in premature ovarian failure because proliferation of germline stem cells improves ovarian reserve and infertility. However, their research has not been verified by animal experiments and in vitro experiments cannot be completely equal with in vivo experiments, so this is not enough. In addition, the target cells their research focuses on are different from ours. These two studies were not sufficient for the treatment of POF with MET, and we did not find other relevant studies, indicating that the study of MET in the treatment of POF especially the relevant mechanisms of action need to be further explored.

In this study, we found that after administering chemotherapy drugs to build POF model animals, the ovaries were in a state of high inflammation and oxidative stress and the polarization of M1 macrophages was disturbed, however, after administration of MET, we observed improvements in endocrine disorders, inflammatory states, and oxidative stress in POF mice. This suggests that MET can alleviate chemotherapy-induced ovarian inflammation and oxidative stress, which may be the reason why MET improves POF. These findings suggest that the administration of MET before or after the administration of chemotherapeutic agents may reduce the incidence of POF in patients who have to receive chemotherapy. In addition, numerous studies have consistently demonstrated that MET primarily exerts its effects through the activation of AMPK^55^. Reports show that the activation of AMPK can activate SIRT1 (histone deacetylase 1)^25^, and PPAR-γ (peroxisome proliferator-activated receptor γ), a nuclear receptor, acts as a transcription factor to inhibit SITR1 gene expression, and the expression of PPAR-γ is inhibited by AMPK^26,27^. Many studies have confirmed the role of this pathway in other diseases and models such as Antrodan can alleviate fatty liver disease through AMPK/AAPR-γ/SIRT1 pathway^56^, Berberine promote adipose tissue remodeling and thermogenesis via AMPK/PPAR-γ /SIRT1 pathway^57^ and so on. However, this pathway has not been studied in the ovary. In our study, KEGG analysis revealed signaling pathways like cell death, immune response, and PPAR pathways were enriched in the POF mice model. Based on the findings from these analyses and previous studies, indirect co-culture of mouse primary GCs and M1 macrophages was performed to explore the mechanism of MET in alleviating ovarian inflammation to improve ovarian damage. In short, we proposed for the first time that MET enhances the anti-inflammatory and anti-oxidative capacity of GCs through the AMPK/PPAR-γ/SIRT1 pathway, ultimately improving GCs' ability to maintain cellular homeostasis, which suggests that AMPK/PPAR-γ-SIRT1 may be a potential target for the treatment of POF.

Although we have done a relatively large amount of study, our work still has several limitations. Firstly, in exploring the mechanism of action of MET, we focused on evaluating its impact on the AMPK/PPAR-γ/SIRT1 signaling pathway, as AMPK is a classical pathway associated with MET^55^. And in a previous study, it has been reported that the impairment of AMPK in the blood of POF patients can be related to the metabolic pathophysiology of POF^58^. Besides, SIRT1 is also very important to GCs and POF^22^. However, MET may protect GCs and improve POF through other pathways as well. Further investigation is needed to determine whether MET affects other signaling pathways involved in the development of POF. Additionally, our mechanistic exploration was limited to cell models only. Thirdly, due to ethical considerations, the protective effects of MET were tested only in mice models of POF, and its extrapolation to humans still has significant limitations. Further clinical and translational research is required before applying our research findings to human patients. Lastly, we only studied the ability of MET to enhance the maintenance of GC homeostasis, and whether it directly affects oocyte function warrants more investigation. All in all, further research is needed to fully understand the role of MET in the treatment of POF and we hope that our study can give new ideas for the treatment of POF.

## Conclusion

Our experiments on animal models have shown that intragastric administration of MET improves ovarian damage induced by chemotherapy in POF mice, by alleviating hormone disruption and reducing inflammation and oxidative stress-related damage. In our in vitro experiments, we aimed to simulate the inflammatory conditions within the ovaries by co-culturing primary GCs with M1 macrophages. We observed that the indirect co-culture of primary GCs with M1 macrophages increased the accumulation of inflammatory factors, oxidative stress, and senescence in primary GCs, and after treatment with MET, the damage caused by M1 supernatant to GCs was alleviated. When verifying the possible pathways involved in the role of MET, we observed that MET improves GCs injury induced by M1 macrophages through the AMPK/PPAR-γ/SIRT1 pathway. In summary, our study demonstrated that the imbalance of macrophage polarization, increased secretion of inflammatory cytokines and accumulation of ROS in the ovaries cause GCs senescence and damage in a chemotherapeutic induced POF mouse model. MET can improve the anti-inflammatory and antioxidant capacity of GCs through AMPK/PPAR-γ/SIRT1 pathway and alleviate POF.

### Supplementary Information

Supplementary Information 1.

Supplementary Information 2.

Supplementary Information 3.

## Data Availability

The datasets used and/or analyzed during the current study available from the corresponding author on reasonable request.

## Abbreviations

AF: Atresia follicle
AMH: Anti-Mullerian Hormone
AMPK: Adenosine monophosphate-activated protein kinase
ARG-1: Arginase-1
BSA: Bovine serum albumin
BUS: Busulfan
CAT: Catalase
CD206: Cluster of differentiation 206
CD86: Cluster of differentiation 86
CL: Corpus luteum
CTX: Cyclophosphamide
CXCL-10: CXCchemokineligand-10
DAPI: 4′,6-Diamidino-2-phenylindole
DMSO: Dimethyl sulfoxide
E2: Estradiol
ELISA: Enzyme-linked immunosorbent assay
FBS: Fetal bovine serum
FSH: Follicle-stimulating hormone
FSHR: Follicle stimulating hormone receptor
GC: Granulosa cell
GPX: Glutathione peroxidase
HCG: Human chorionic gonadotropin
HE: Hematoxylin and eosin
IL-10: Interleukin-10
IL-6: Interleukin-6
INFOS: Inducible nitric oxide synthase
LPS: Lipopolysaccharide
MET: Metformin
PBS: Phosphate buffer saline
PF: Primary follicle
PMSG: Pregnant mare serum gonadotropin
POF: Premature ovarian failure
PPAR-γ: Peroxisome proliferator-activated receptor γ
qPCR: Quantitative real-time PCR
ROS: Reactive oxygen species
SASP: Senescence associated secretory phenotype
SF: Secondary follicle
SIRT1: Sirtuin 1
SOD: Superoxide dismutase
TBST: Tris buffered saline with tween
TGFβ: Transforming growth factor-β
TNF-α: Tumor necrosis factor α
WB: Western blot
