# Supplementary material for: Metformin protects ovarian granulosa cells in chemotherapy-induced premature ovarian failure mice through AMPK/PPAR-γ/SIRT1 pathway
Source: Sci Rep. 2024 Jan 16;14:1447. doi: 10.1038/s41598-024-51990-z (PMC10791659; doi:10.1038/s41598-024-51990-z)
Supplement: Supplementary file 1 — Supplementary Information 1. [file 41598_2024_51990_MOESM1_ESM.pdf]

南昌大学
NANCHANG UNIVERSITY

IACUC

编号 Approval No. NCULAE-20221130010

南昌大学动物实验伦理审查批准书

Nanchang University Approval for Research Involving Animals

<table>
<tr><td>项目主持人
Principal Investigator (PI)</td><td>Yuxin Yang、Xiangting Tang</td><td>院系（单位）
Department (Affiliation)</td><td colspan="3">Jiangxi Medical College, Nanchang University</td></tr>
<tr><td>项目名称
Protocol Title</td><td colspan="5">Metformin protects ovarian granulosa cells in chemotherapy-induced premature ovarian failure mice through AMPK/PPAR-γ/SIRT1 pathway</td></tr>
<tr><td colspan="2">项目来源和批号
(Project Source & Number)</td><td colspan="4">the National Natural Science Foundation of China [grant number 81860263] and the Natural Science Foundation of Jiangxi Province of China [grant number 20192BAB205119]</td></tr>
<tr><td rowspan="4">使用动物情况
Animal Requirements</td><td>动物来源 Source</td><td>GemPharmatech</td><td>品种品系 Species or Strains</td><td colspan="2">KM mice</td></tr>
<tr><td colspan="5">数量 Quantity 36 只（♀ 36 只；♂ 0 只）</td></tr>
<tr><td colspan="5">动物实验设施许可证编号 Name and certificate number of the facility
☐ SYXK（赣）2021-0001 √ SYXK（赣）2021-0004</td></tr>
<tr><td>计划开始日期（Proposed Date of Commencement）</td><td>2021Y7M1D</td><td>计划结束日期（Proposed Date of Completion）</td><td colspan="2">20227M1D</td></tr>
<tr><td>审查项目
Considerations Ethical</td><td colspan="5">1. 该项目经过实验动物福利伦理委员会审核，符合动物保护、动物福利和伦理原则，符合国家实验动物福利伦理的相关规定。（The animal use protocol listed below has been reviewed and approved by the Animal Ethical and Welfare Committee.）
2. 申请人资格和所使用动物的品种品系、质量等级、规格是否合适，能否通过改良设计方案或用高质量的动物来减少所用动物的数量？（Are the qualification of applicant, species or strains, grade and specifications of animals suitable? Could the quantity of animals be reduced by improving the study design or using high quality animals?）
3. 实验操作中是否善待动物，包括合理的实验终点，麻醉方案，不麻醉的理由及减少相应动物痛苦的措施，实验结束动物的处理，动物安乐死方案等。（Appropriate animal care and handling throughout the experiment, including a scientific sound endpoint; anesthetics, analgesics, sedatives or tranquilizers that are to be used; explanation for any procedure cause unrelieved pain or distress; disposition of animals at end of study; and euthanasia criteria and method.）
4. 是否使用对人体或环境有害试剂、有潜在感染性试剂及放射性物质；是否使用遗传修饰试剂；是否进行遗传操作及相应的防护措施。（Are the materials to be used harmful or toxic? Are there any radioactive agents, infectious agents, genetic modified agents, and the genetic manipulation to be used in the experiment? If yes, the safety measures should be specified.）</td></tr>
<tr><td colspan="2">福利伦理委员会审批意见
Approval opinion of Committee

☑批准 Agree
☐不批准 Disagree</td><td colspan="4">签名(盖章):
Name（Stamp）
Date:
[Stamp: 南昌大学实验动物福利伦理委员会 3601020230917]</td></tr>
</table>
