# Supplementary material for: Metformin protects ovarian granulosa cells in chemotherapy-induced premature ovarian failure mice through AMPK/PPAR-γ/SIRT1 pathway
Source: Sci Rep. 2024 Jan 16;14:1447. doi: 10.1038/s41598-024-51990-z (PMC10791659; doi:10.1038/s41598-024-51990-z)
Supplement: Supplementary file 2 — Supplementary Information 2. [file 41598_2024_51990_MOESM2_ESM.docx]

**Supplementary material:**


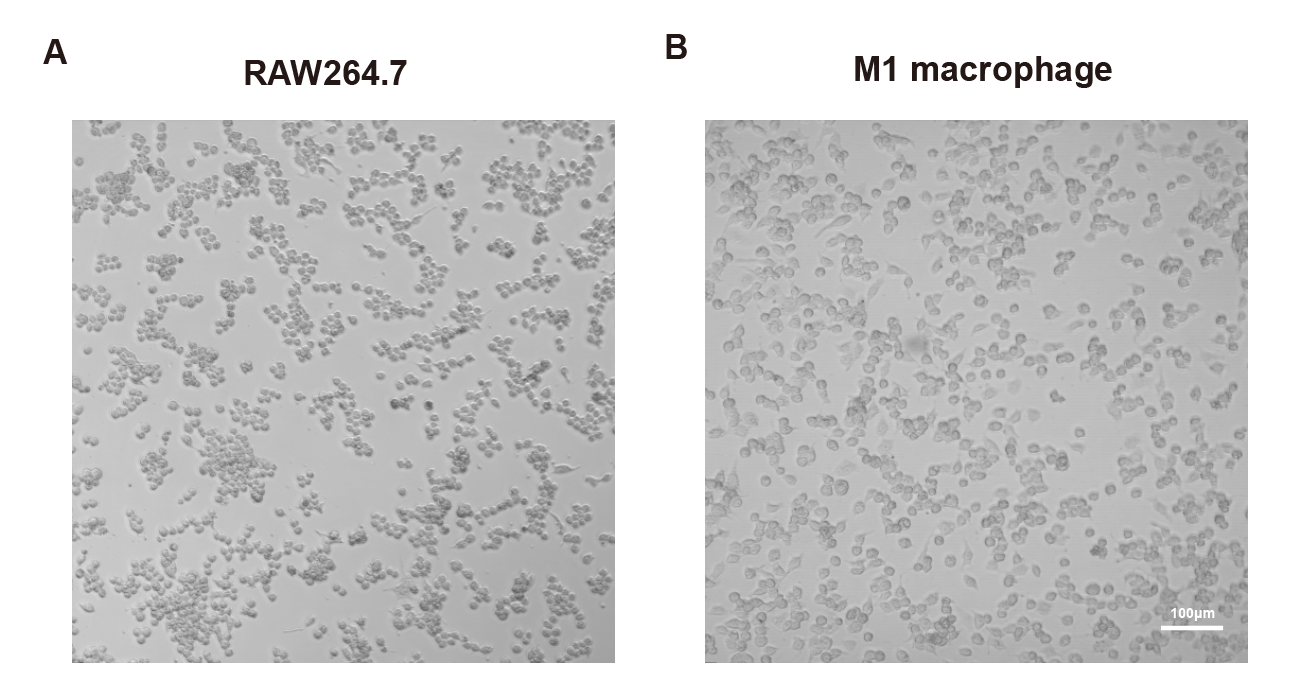
Morphological changes of RAW264.7 cell line after polarization into M1 macrophages, giving rise to pseudopodia from the round. A: RAW264.7 cells, round, agglomerated, weakly adherent, semi-suspended; B: M1 macrophage, extending pseudopodia, irregular, adherent growth, strong adherence, slightly larger size.
