# Supplementary material for: Metformin protects ovarian granulosa cells in chemotherapy-induced premature ovarian failure mice through AMPK/PPAR-γ/SIRT1 pathway
Source: Sci Rep. 2024 Jan 16;14:1447. doi: 10.1038/s41598-024-51990-z (PMC10791659; doi:10.1038/s41598-024-51990-z)

Multi-channel

Raw data Figure1——WB-RAW data of Figure3 (CON-POF-POF+MET)


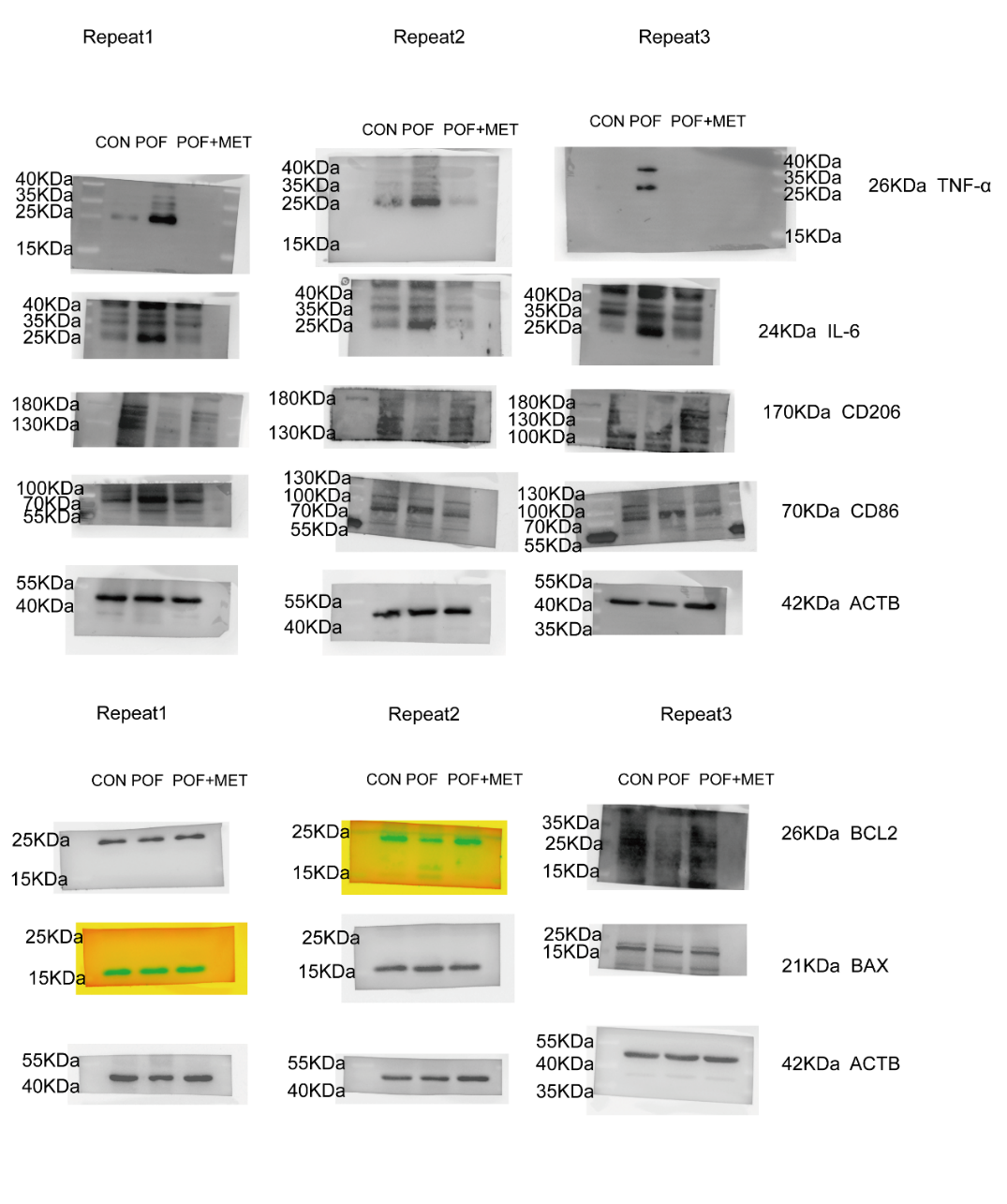


Note: We declare and ensure that our WB data comply with the digital image and integrity policies. All images were unchanged (Only cropped and all images are scaled at a fixed width/height ratio)). The PVDF membranes were cut before hybridization with a single specific target antibody during blotting for better imaging. For some antibodies with poor specificity and more miscellaneous bands, whether they were the target protein was judged according to the prestained protein marker. If the imaging effect is too poor to distinguish two target proteins that are very close to each other, they can only be repeated once and incubated separately. Two channels were used for the exposure, one for the marker (which can show the shape of the PVDF membrane and the marker's location) and one for the target band(if the antibody is effective or the expression of the target protein is high, after incubation with specific antibody, only one clear target band can be seen in black). After the exposure, multi-channel synthesis was directly performed on Image lab6.1. It was then exported to TIFF format for results display and gray value analysis (using image J), and the alignment and annotation were completed on AI. Most of the dual-channel composite images were turned gray, but some markers could not see clearly after turning gray, so the RGB format was retained. Red boxes are marked for the bands presented in the manuscript.

Raw data Figure2——WB-RAW data of Figure4 (CON-co-M0-co-M1; CON-LPS)


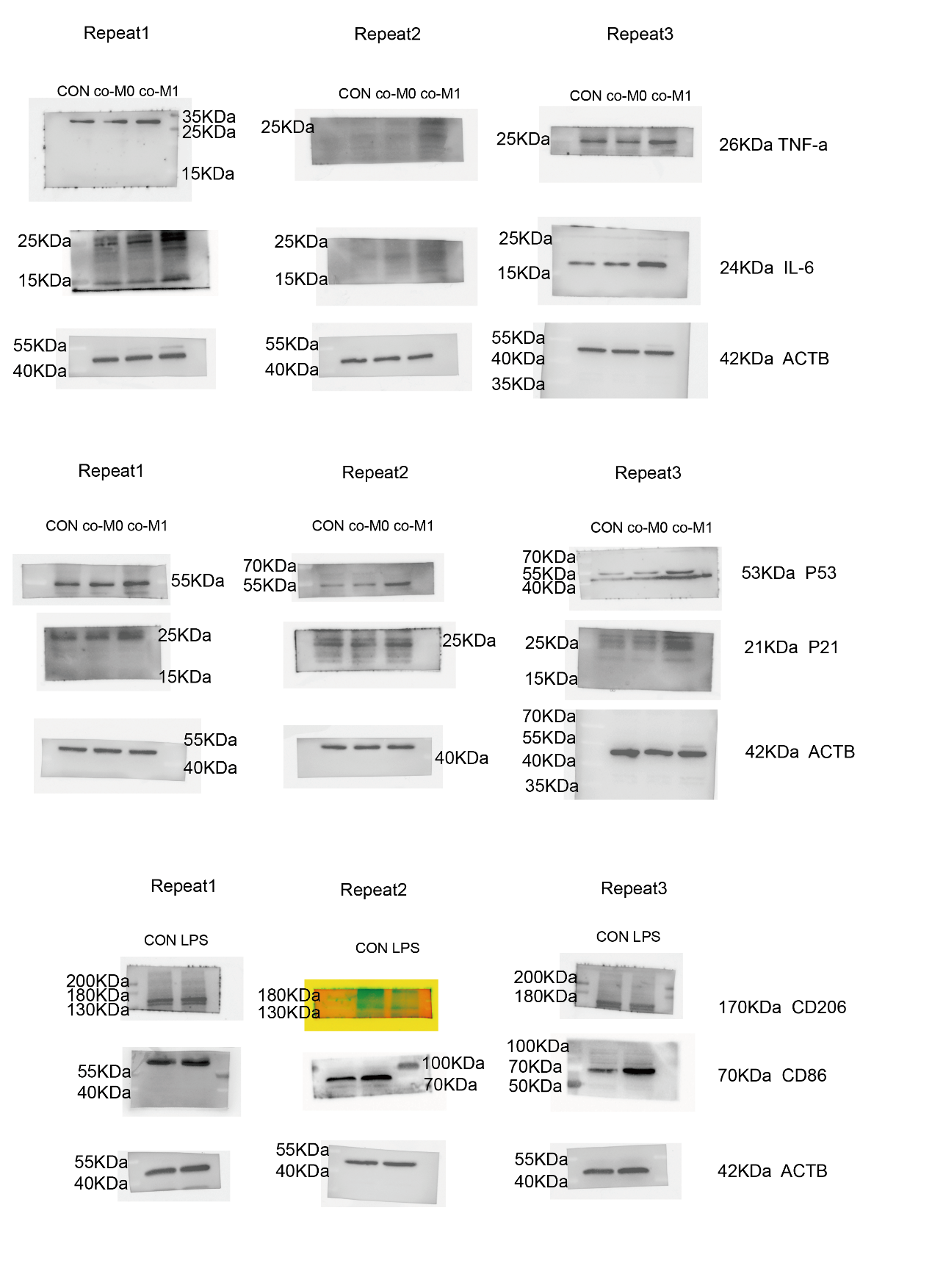


Raw data Figure3——WB-RAW data of Figure5 (CON-co-M1-co-M1+MET-MET)


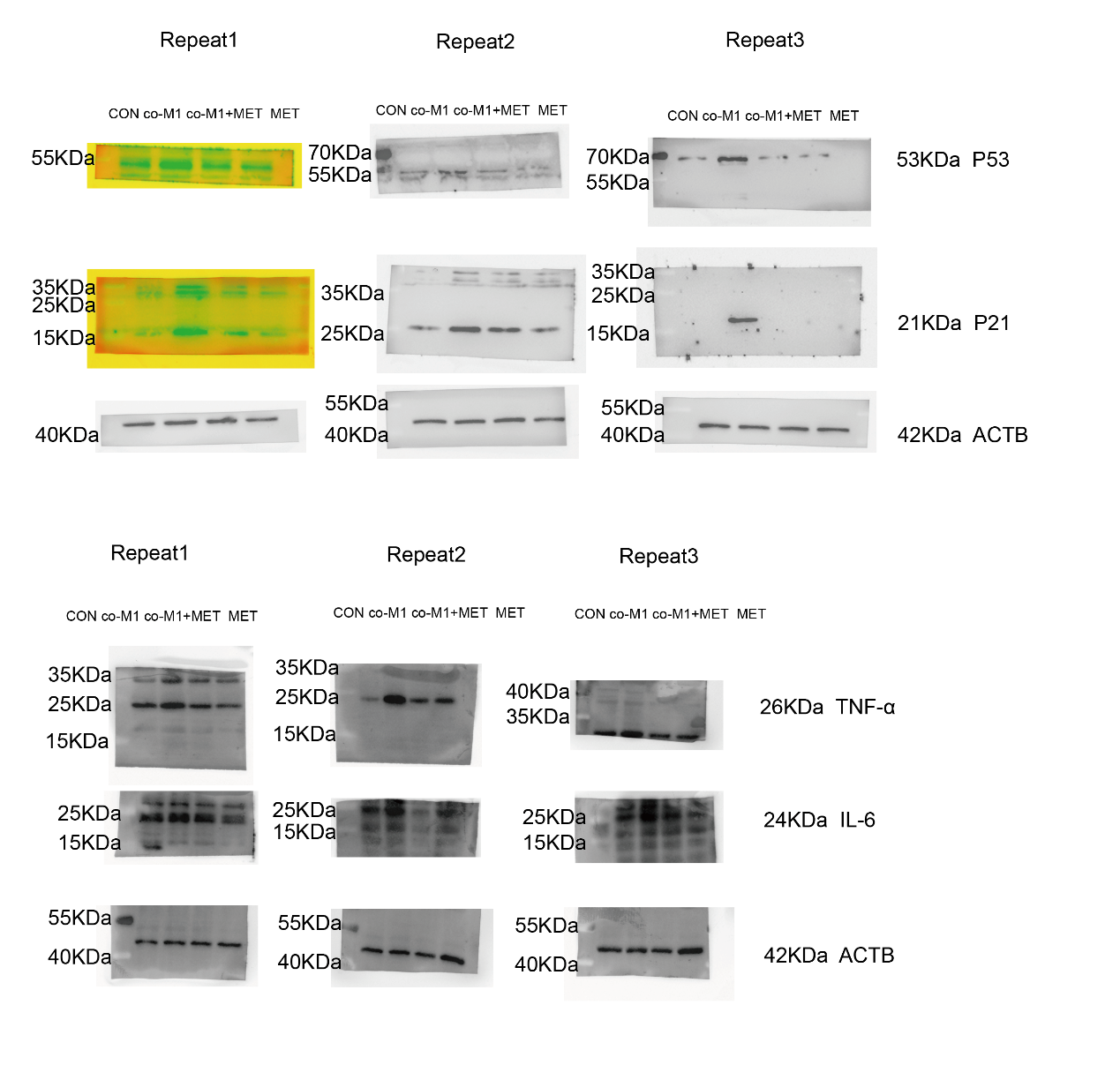


Raw data Figure4——WB-RAW data of Figure6 (CON-co-M0-co-M1; CON-co-M1-co-M1+MET-MET;CON-co-M1-co-M1+MET-co-M1+MET+EX527-MET-EX527)


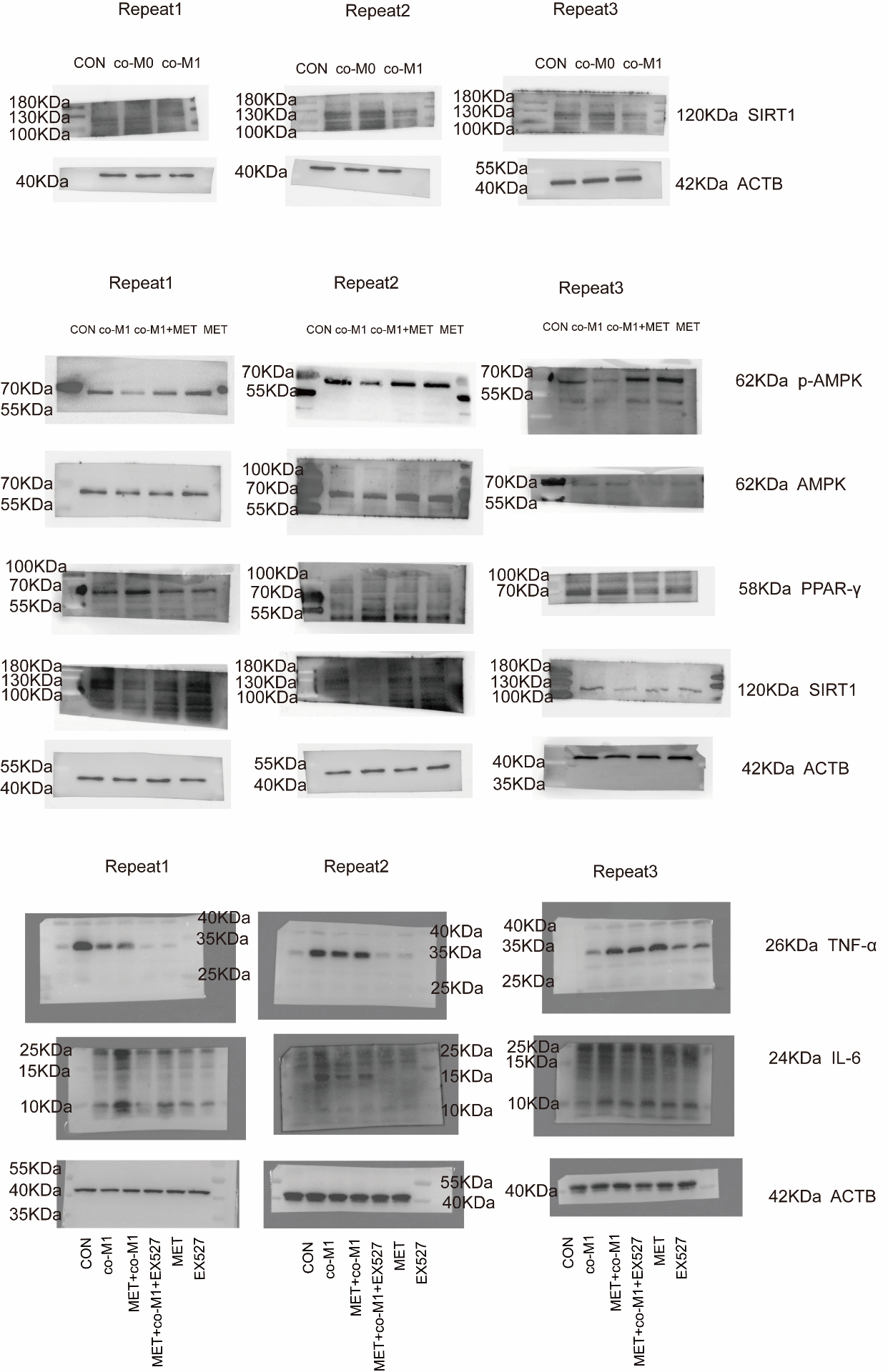


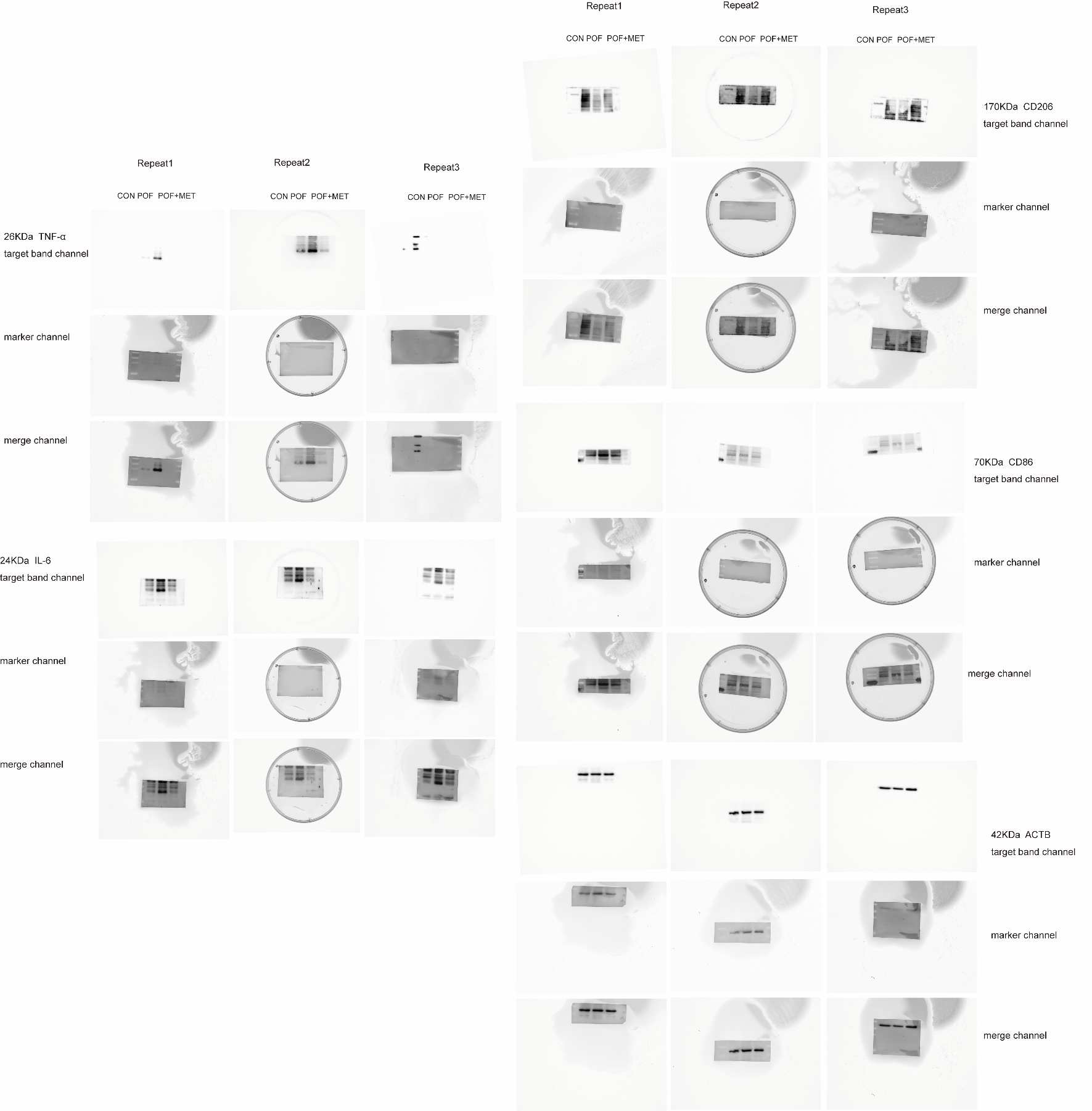
Single-channel (two channels as well as merge, all images are in tiff format exported from the original data) WB-RAW data of Figure3 (CON-POF-POF+MET)


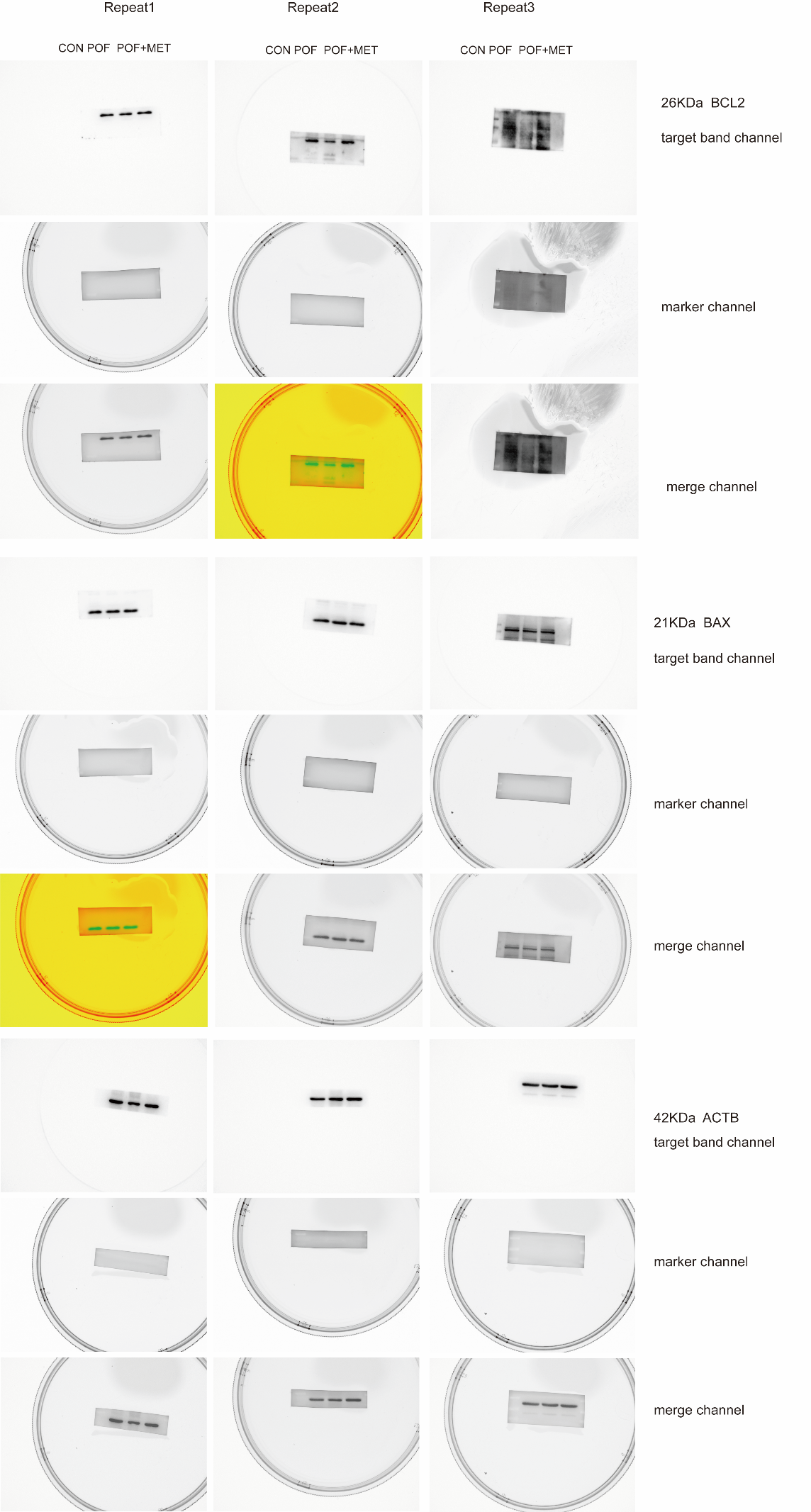


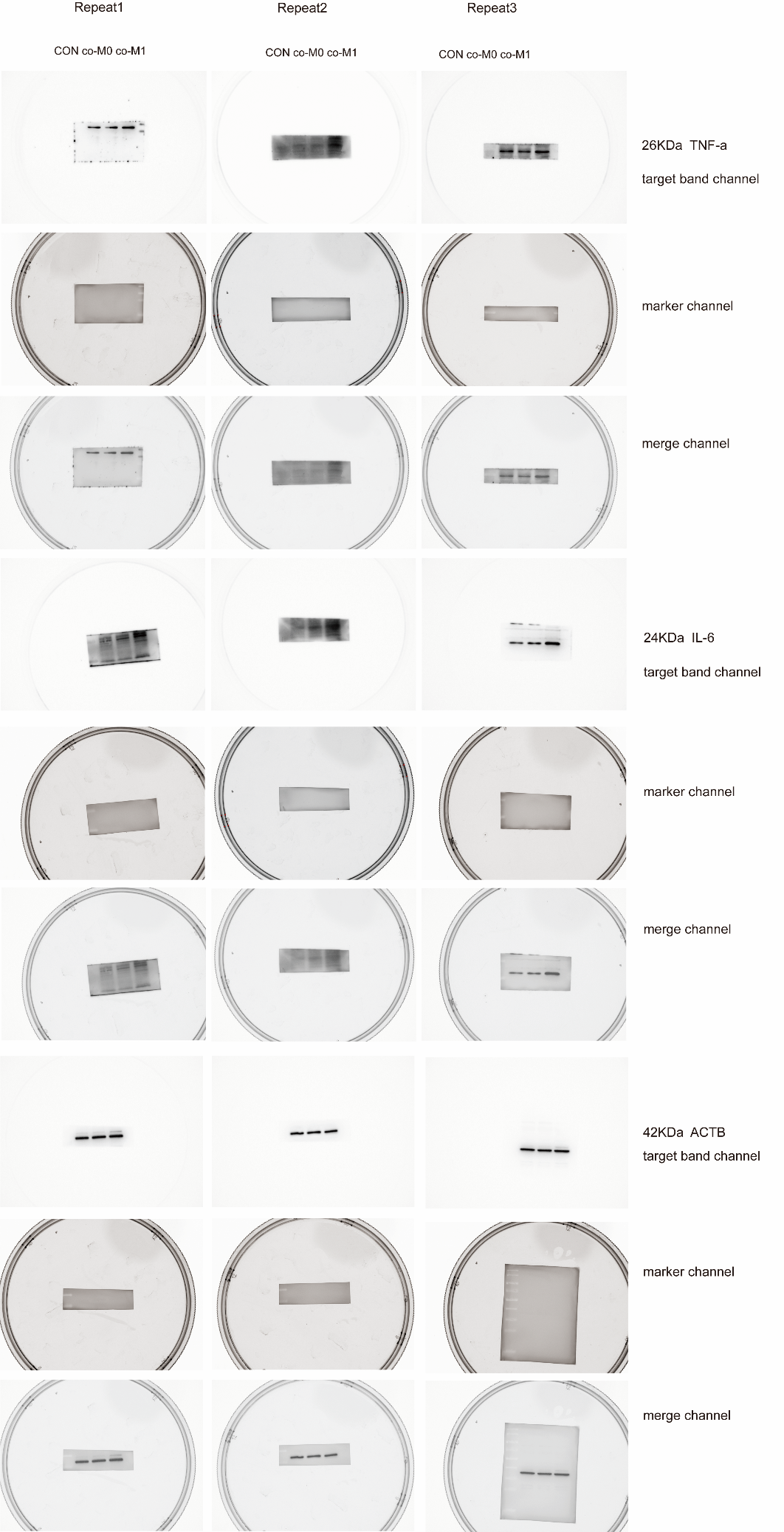
Single-channel WB-RAW data of Figure4 (CON-co-M0-co-M1; CON-LPS)


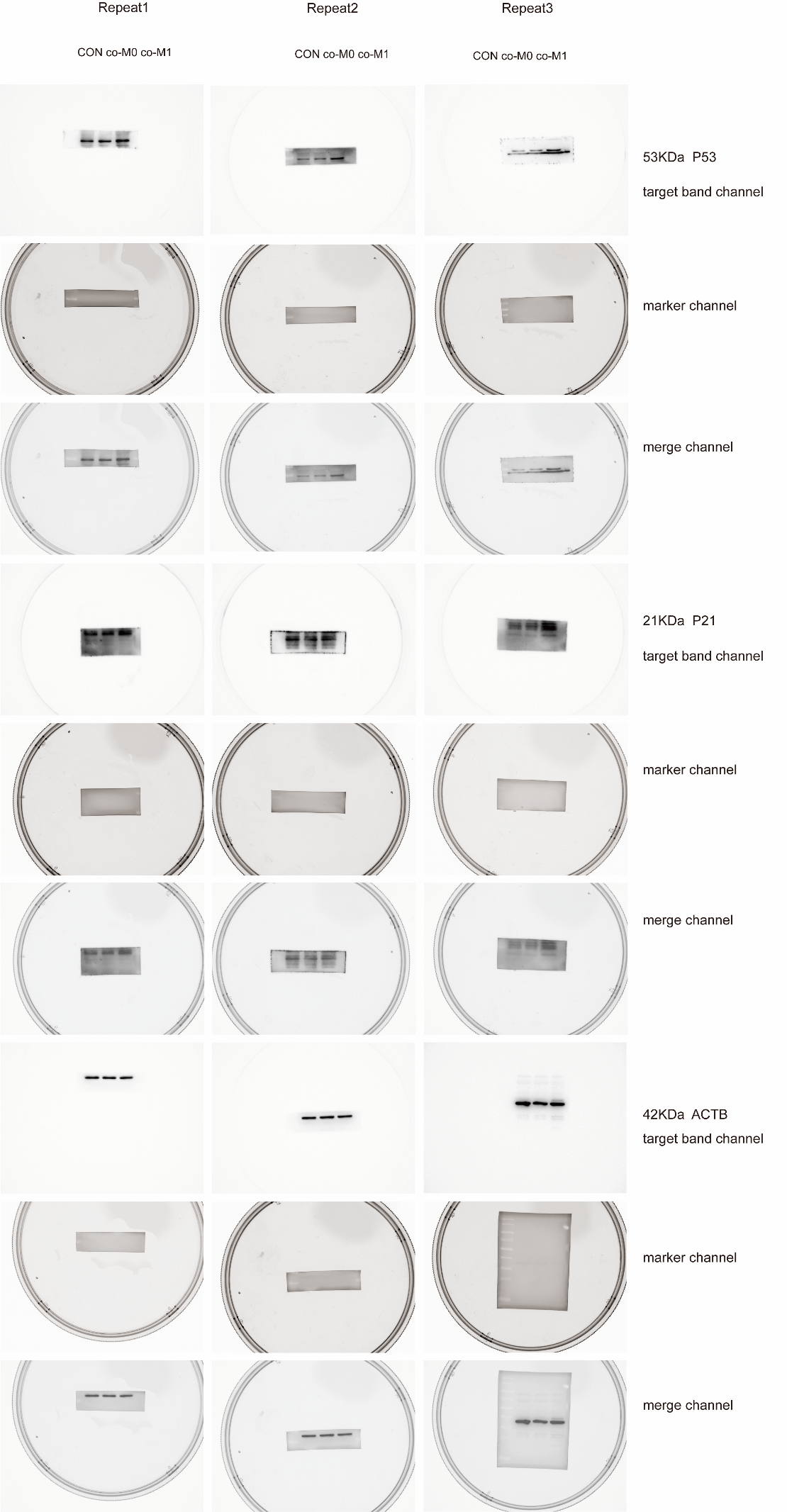

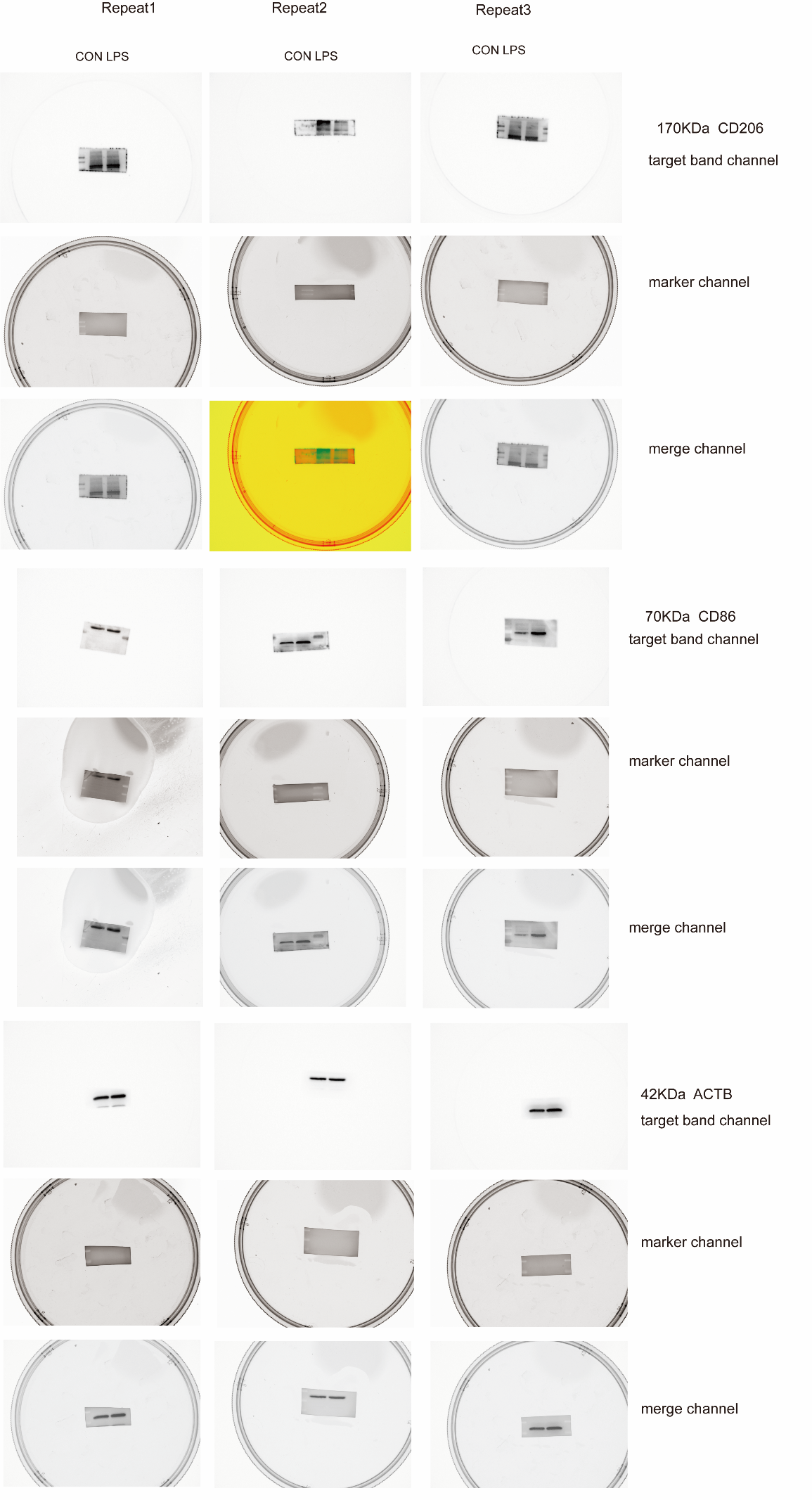


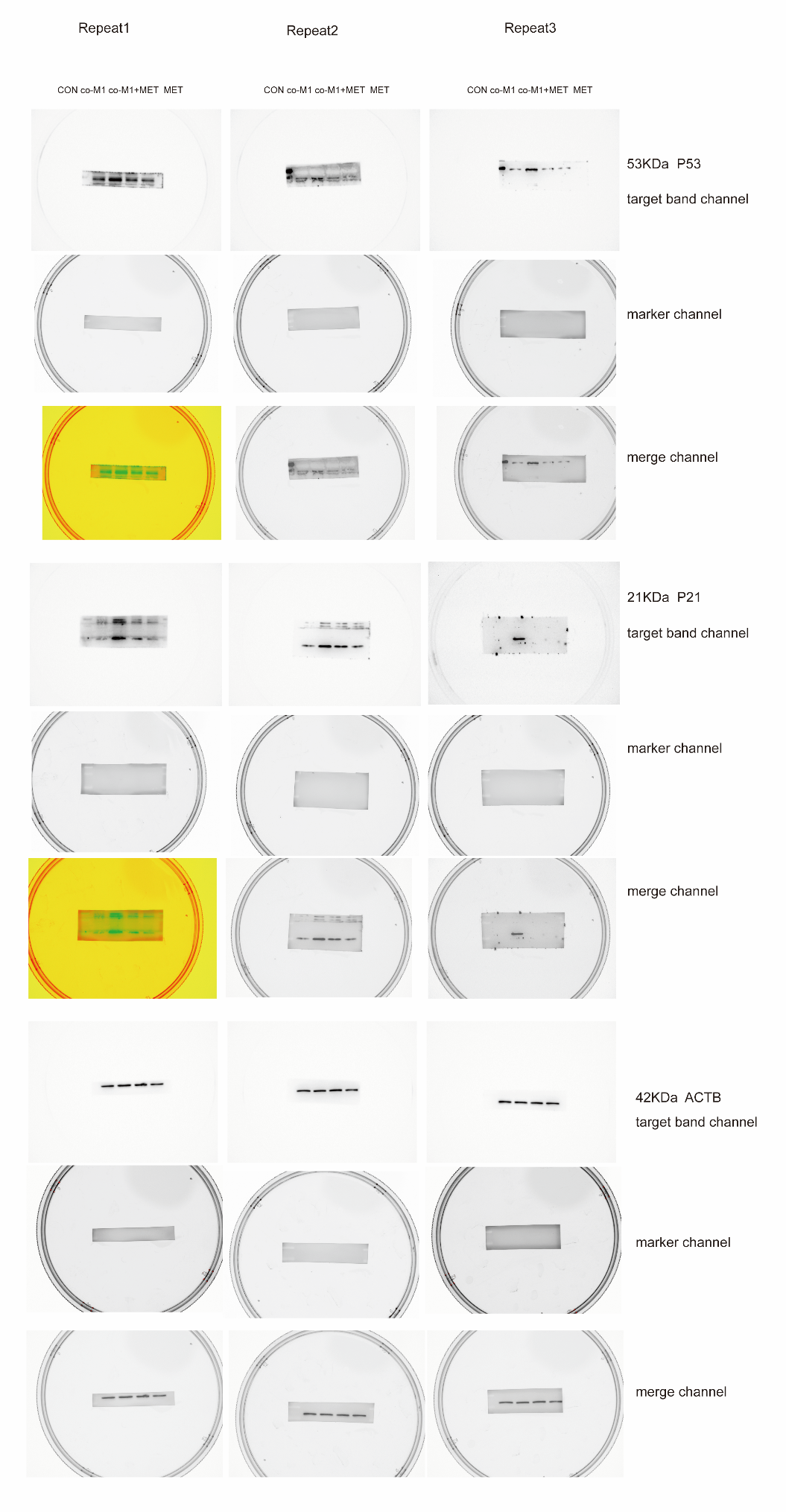
Single-channel WB-RAW data of Figure5 (CON-co-M1-co-M1+MET-MET)


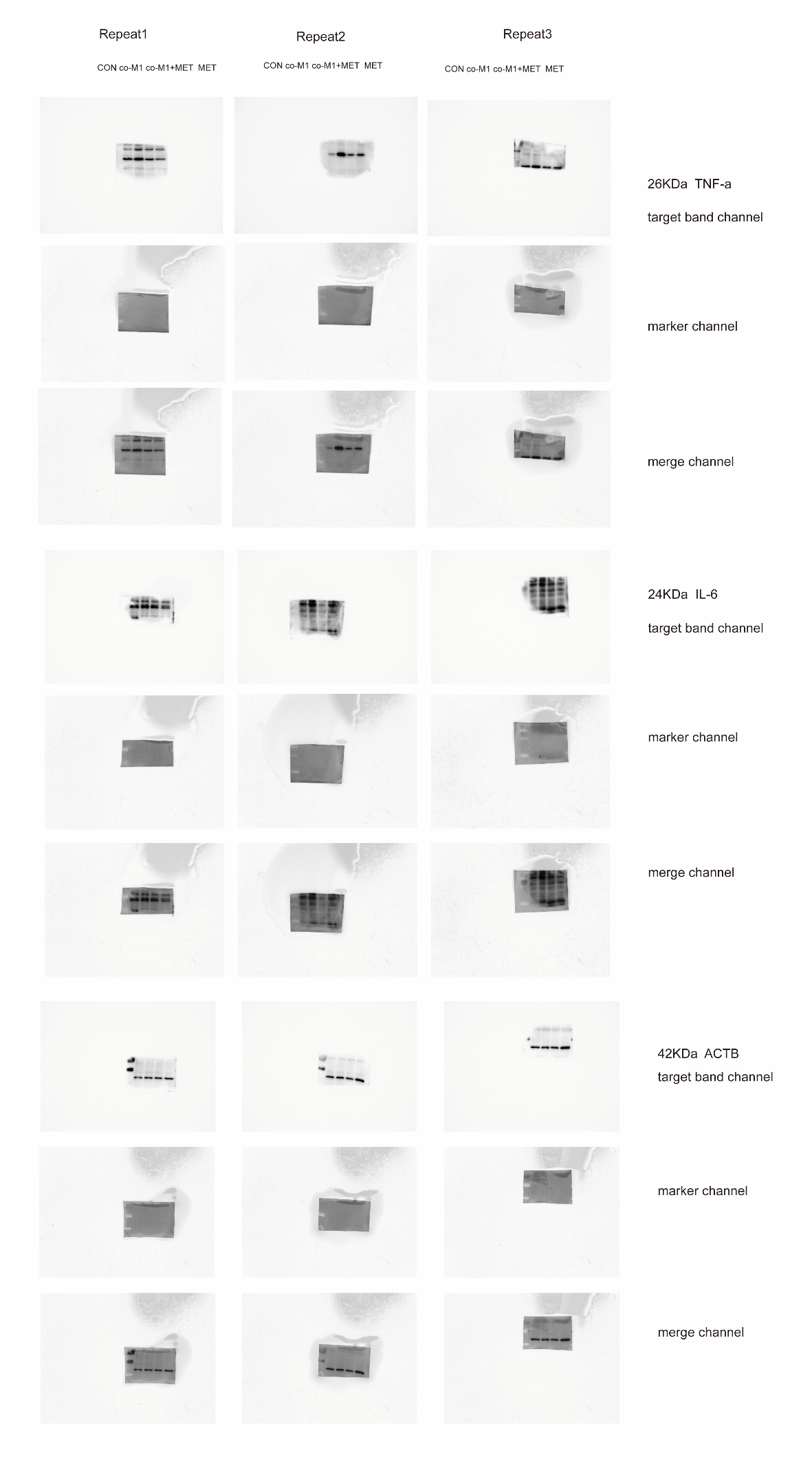


Single-channel WB-RAW data of Figure6 (CON-co-M0-co-M1; CON-co-M1-co-M1+MET-MET; CON-co-M1-co-M1+MET-co-M1+MET+EX527-MET-EX527)


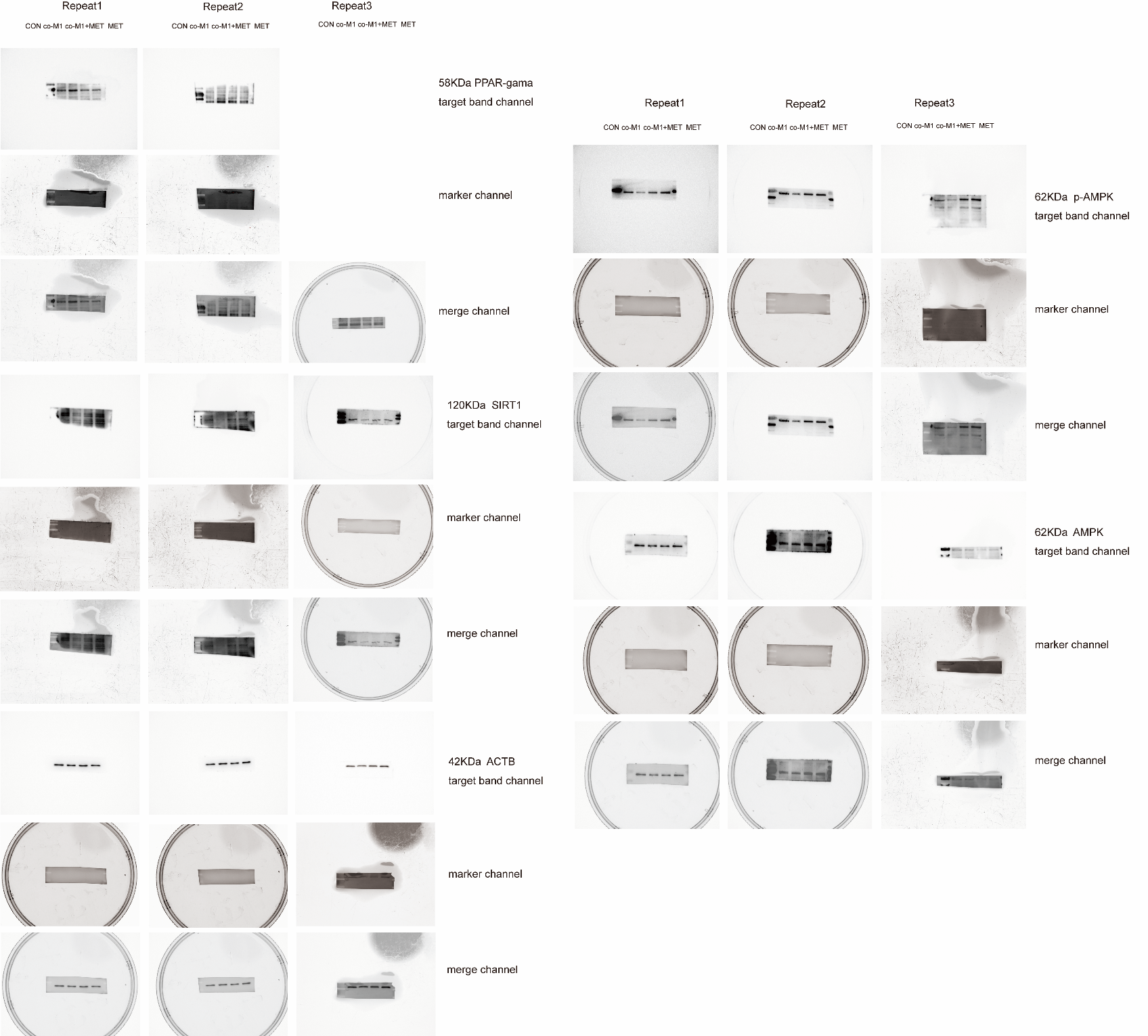
Description: The file under the single channel copy of PPAR-γ repeat 3 was corrupted (The original data on the computer instrument has been deleted because it is too long), and the image tiff format was not saved at that time, so only the merged image is left in this set of images. However, this does not affect the analysis after data collection.


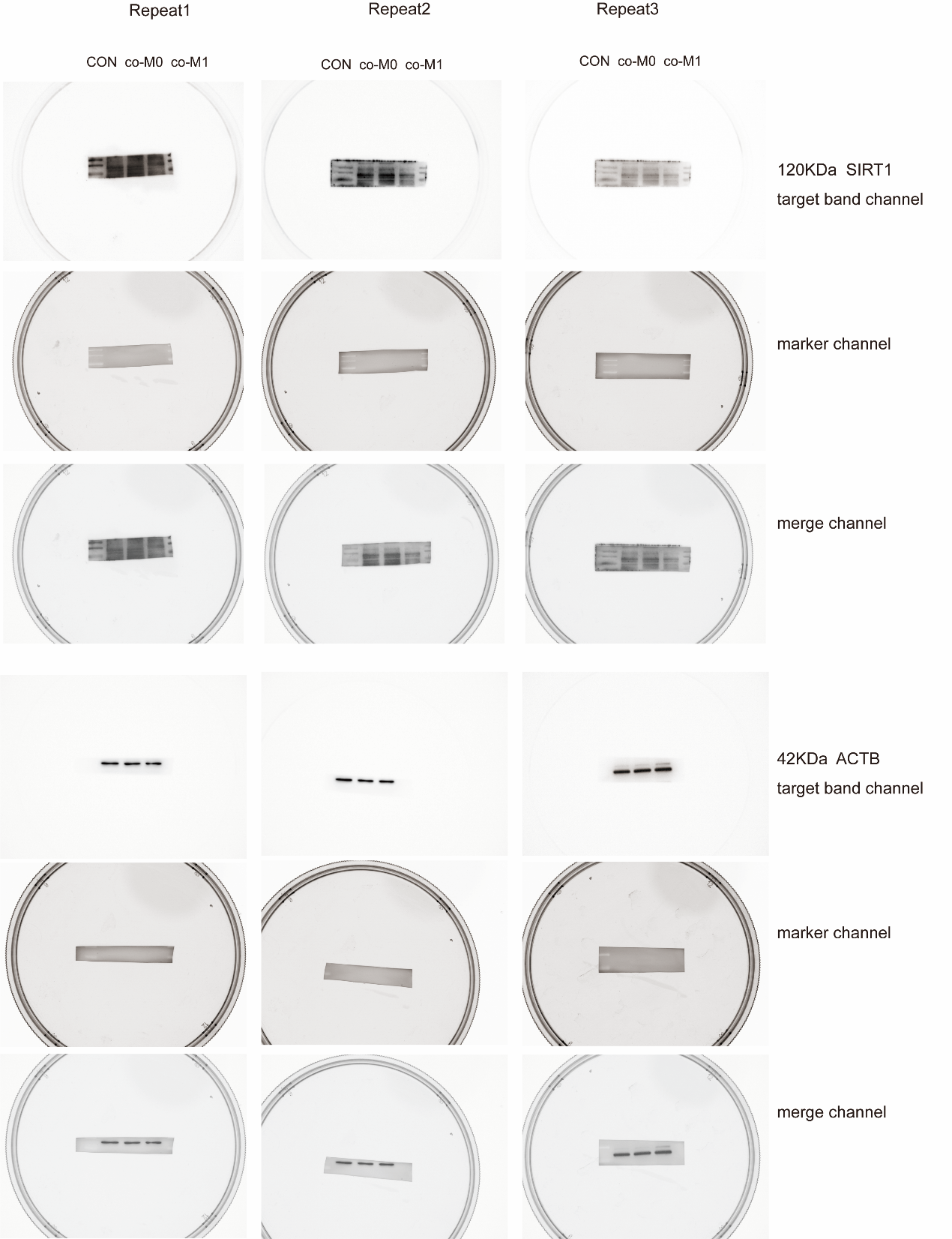

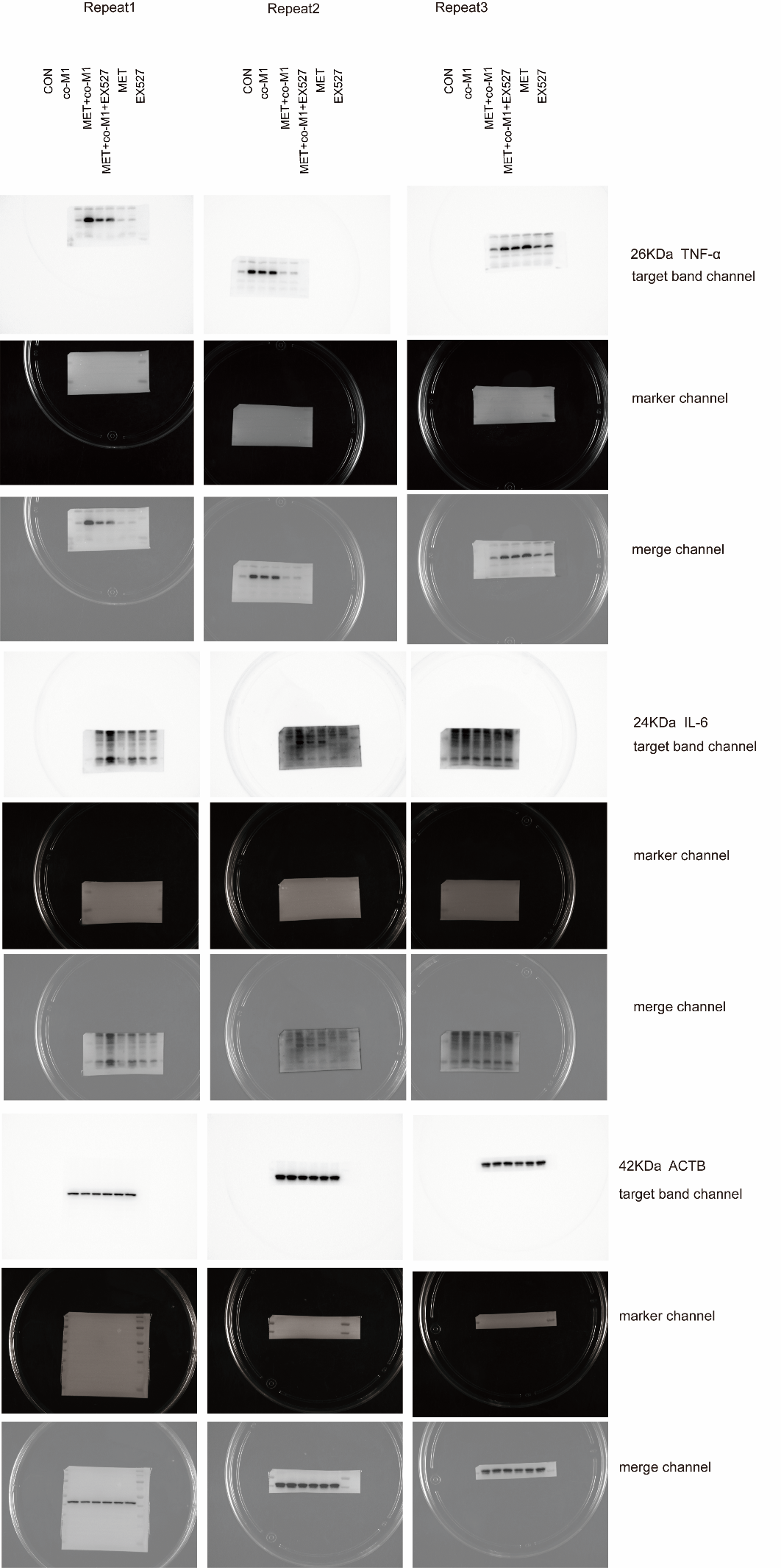

Supplement: Supplementary file 3 — Supplementary Information 3. [file 41598_2024_51990_MOESM3_ESM.docx]
